# Supplementary material for: Analysis of a shark reveals ancient, Wnt-dependent, habenular asymmetries in vertebrates
Source: Nat Commun. 2024 Nov 25;15:10194. doi: 10.1038/s41467-024-54042-2 (PMC11589584; doi:10.1038/s41467-024-54042-2)
Supplement: Supplementary file 1 — Supplementary Information [file 41467_2024_54042_MOESM1_ESM.pdf]

Analysis of a shark reveals ancient, Wnt-dependent, habenular asymmetries in vertebrates

SUPPLEMENTARY MATERIAL

List of Supplementary Figures and Tables:

Supplementary Figure 1. Functional annotation of left- and right-enriched genes identified by transcriptomic analysis.

Supplementary Figure 2. Expression profiles of left- or right-enriched genes showing regionalized ISH (*in situ* hybridization) profiles in stage 31 catshark habenulae.

Supplementary Figure 3. Expression of *ScSox1*, *ScPde1a*, *ScKctd12b*, *ScEnpp2* and *ScProx1* along the antero-posterior axis of stage 31 catshark habenulae.

Supplementary Figure 4. Expression of *ScSox1*, *ScPde1a*, *ScKctd12b*, *ScEnpp2* and *ScProx1* along the dorso-ventral axis of stage 31 catshark habenulae.

Supplementary Figure 5. Subdomain organization of juvenile catshark habenulae.

Supplementary Figure 6. Expression of mouse orthologs of catshark Left-LHb, MHb and Right-LHb markers.

Supplementary Figure 7. Subdomain organization of habenulae in the elephant shark *Callorhinchus milii*.

Supplementary Figure 8. Subdomain organization of habenulae in the reedfish *Erpetoichthys calabaricus*.

Supplementary Figure 9. Subdomain organization of habenulae in the spotted gar *Lepisosteus oculatus*.

Supplementary Figure 10. Subdomain organization of habenulae in the lungfish *Protopterus annectens*.

Supplementary Figure 11. Subdomain organization of habenulae in the Western clawed frog *Xenopus tropicalis.*

Supplementary Figure 12. Heterogeneity of β-catenin distribution in the catshark medial habenula at stage 31.

Supplementary Figure 13. Phenotypes observed along the antero-posterior axis of catshark stage 31 habenulae following IWR-1 treatment.

Supplementary Figure 14. IWR-1 treatment at stage 29 has no effect on *ScPde1a* and *ScEnpp2* expression in developing catshark habenulae.

Supplementary Figure 15. Phenotypes observed along the antero-posterior axis of catshark stage 31 habenulae following double SB-505124 and IWR-1 treatment.

Supplementary Figure 16. Spatial and temporal regulation of progenitor cell cycle exits in the developing catshark habenulae.

Supplementary Figure 17. Asymmetric β-catenin signals persist in juvenile catshark habenulae.

Supplementary Figure 18. Maximum likelihood phylogenetic trees showing gene content and relationships within the vertebrate *Pcdh10/10l/17/18/19* (a), *Sox1/2/3/19* (b), *Ntng1/2* (c), *Prox1/2* (d), and *Kctd8/12/16* (e) gene families.

Supplementary Table 1. Expression characteristics of mouse and zebrafish orthologs of markers of the main habenular territories identified in the catshark.

Supplementary Table 2. Asymmetry phenotypes in the lateral habenulae of control and IWR-1-treated catshark embryos.

Supplementary Table 3. Asymmetry phenotypes in the lateral habenulae of SB-505124- and SB-505124-+IWR-1-treated catshark embryos.

Supplementary Table 4. Expression of lamprey (*Petromyzon marinus*) members of the NCBI or Ensembl identifiers *Pcdh10/10l/17/18/19*, *Sox1/2/3/19*, *Ntng1/2*, *Prox1/2*, and *Kctd8/12/16* gene families in habenula, pineal and parapineal cell clusters, as inferred from the single nuclei RNA-seq analysis of the lamprey brain.

a

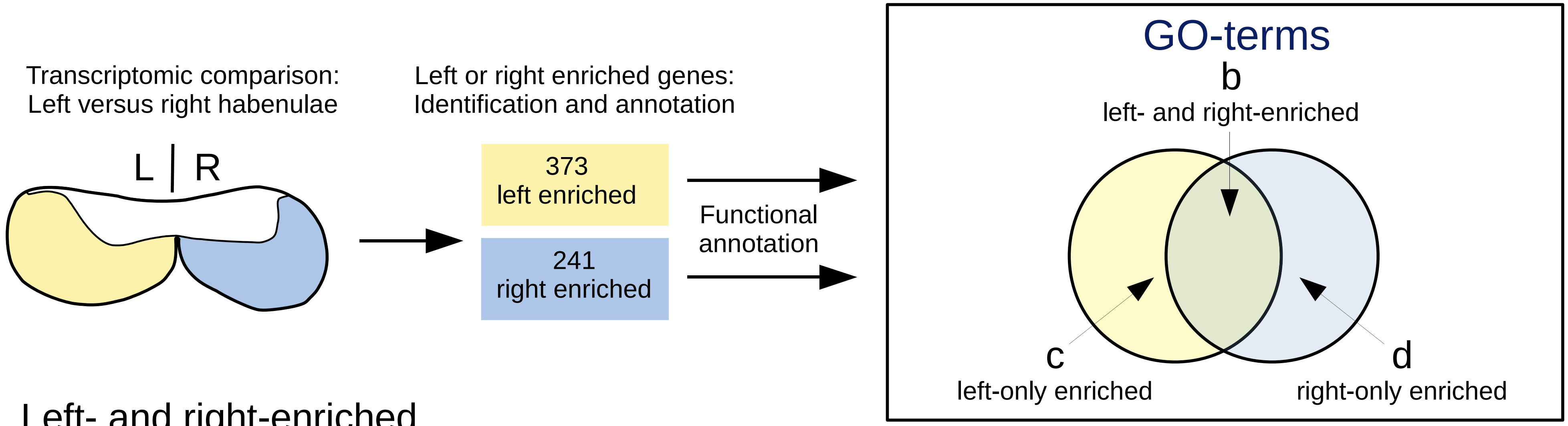

b Left- and right-enriched

CENTRAL NERVOUS SYSTEM		
• neuron differentiation	7.3E-10	3.9E-05
• neurogenesis	2.3E-08	1.5E-06
• neuron projection guidance	1.6E-04	2.7E-03
• neuron projection development	2.7E-06	4.1E-04
• synaptic signaling	6.5E-10	3.5E-08
• synapse organization	8.0E-07	8.3E-03
• regulation of trans-synaptic signaling	7.1E-09	3.0E-05
• regulation of membrane potential	7.4E-04	6.1E-03

ORGANISMAL RESPONSES		
• regulation of locomotion	3.1E-03	6.2E-06
• sensory perception of pain	2.8E-02	6.4E-03
**SIGNALING PATHWAYS**		
• cell-cell signaling by Wnt	1.8E-02	4.9E-02
• glutamate receptor signaling pathway	1.3E-07	3.9E-02
• neuropeptide signaling pathway	2.0E-04	4.8E-06
• dopamine secretion	8.9E-04	2.7E-02

c Left-only enriched

ORGANISMAL RESPONSES	
• sensory perception of mechanical stimulus	4.1E-04
• feeding behavior	2.7E-02
• sleep	4.9E-02
**SIGNALING PATHWAYS**	
• adenylate cyclase-modulating GPCR	2.3E-05
• acetylcholine receptor	1.5E-03
• G protein coupled glutamate receptor	1.5E-03
• ionotropic glutamate receptor	6.8E-03

d Right-only enriched

ORGANISMAL RESPONSES	
• learning or memory	1.3E-03
• response to amphetamine	2.7E-03
• cognition	4.5E-03
• regulation of behavioral fear response	9.8E-03
• behavioral response to nicotine	9.6E-03
**SIGNALING PATHWAYS**	
• opiod receptor	2.4E-02
• phospholipase C-activating GPCR	4.8E-02

Supplementary Figure 1

See legend on next page

Supplementary Figure 1. Functional annotation of left- and right-enriched genes identified by transcriptomic analysis. a Scheme showing the pipeline used for the functional annotation of genes identified by the transcriptomic comparison between left and right catshark habenulae. **b-d** Lists of over-represented GO- (Gene Ontology) terms retrieved from both left- and right-enriched gene lists (b), only from the list of left-enriched genes (c) or only from the list of right-enriched genes (d). For each term, the corresponding p-value (shaded in yellow for left-enriched genes and in blue for right-enriched genes) is indicated. Abbreviations: GPCR, G protein coupled receptor; L, left; R, right.

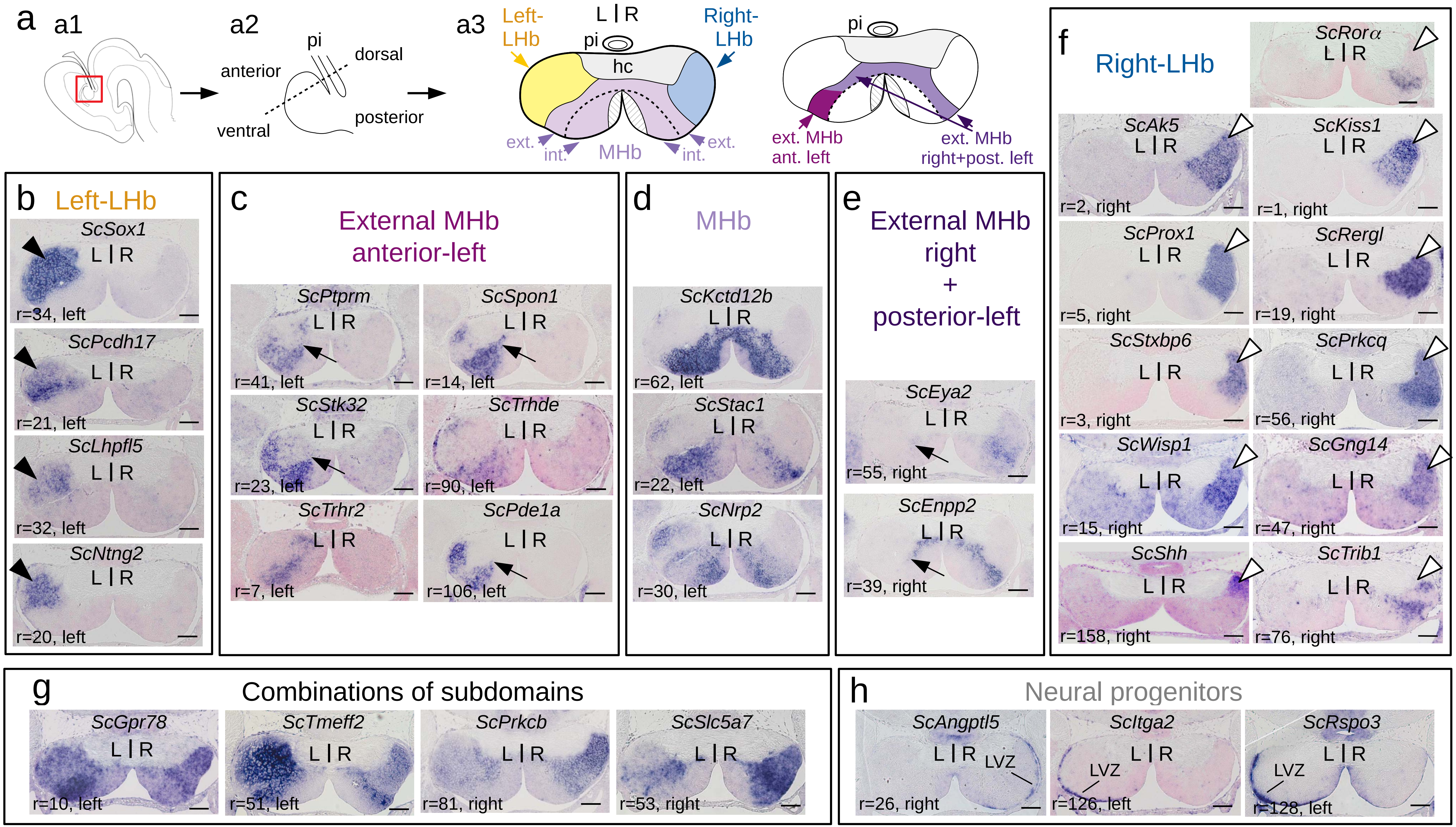

Supplementary Figure 2

See legend on next page

Supplementary Figure 2. Expression profiles of left- or right-enriched genes showing regionalized ISH profiles in stage 31 catshark habenulae. **a** Schemes showing a left lateral view of a stage 31 embryonic brain with habenulae boxed in red (a1), a lateral left view of habenulae with a dotted line indicating the transverse section plane used (a2) and the subdomain organization observed on a section at a medial level (a3). The dashed line in (a3) delimits the external and internal components of the medial habenula. Color code: yellow, Left-LHb; light purple, MHb; blue, Right-LHb; magenta, anterior left external MHb; dark purple, right plus posterior left external MHb; hatched, proliferative pseudo-stratified neuroepithelium. **b-h** transverse sections following ISH with probes for Left-LHb markers (b), anterior left external MHb markers (c)**,** MHb markers (d)**,** right+posterior left external MHb markers (e), Right-LHb markers (f)**,** genes expressed in combinations of LHb and MHb territories (g) and markers of LVZ neural progenitors (h). The expression profile of *ScRor*α, which was not retrieved by the transcriptomic analysis, is also shown in (f). Dorsal is to the top for all sections. For each gene retrieved from the transcriptomic analysis, the rank (r), based on q-value in the list of asymmetrically expressed genes, and the laterality of the enrichment are indicated at the bottom left of the panel. Black and white arrowheads in (b,f) respectively point to Left- and Right-LHb labeled territories, thin arrows in (c,e) point to the boundary between complementary anterior and posterior territories within the left external MHb. Abbreviations: ant., anterior; post., posterior; ext., external; int., internal; LVZ, lateral ventricular zone; LHb, lateral habenula; MHb, medial habenula; hc, habenular commissure; L, left; R, right; pi, pineal. Scale bar=100μm.

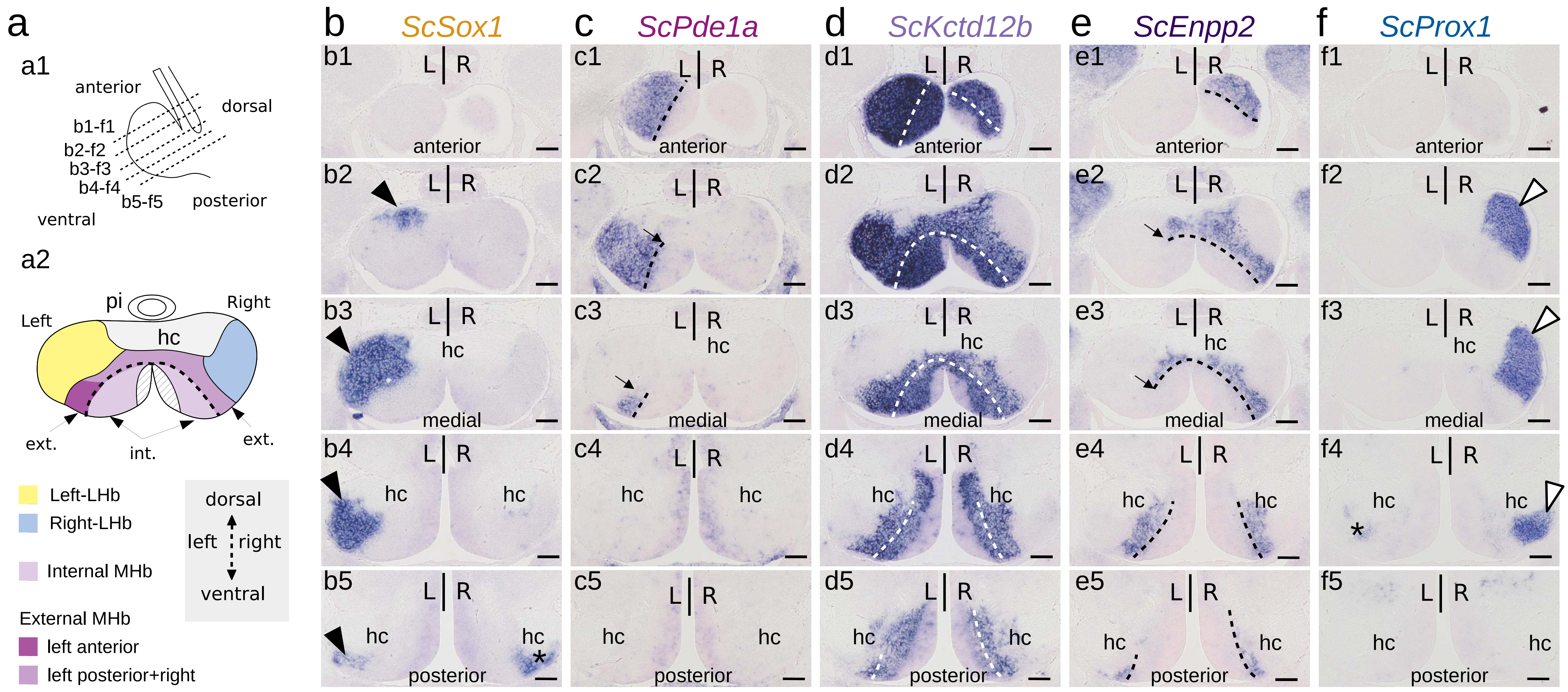

Supplementary Figure 3

See legend on next page

Supplementary Figure 3. Expression of *ScSox1*, *ScPde1a*, *ScKctd12b*, *ScEnpp2* and *ScProx1* along the antero-posterior axis of stage 31 catshark habenulae. a Schemes showing a left lateral view of stage 31 catshark habenulae, with transverse section planes indicated by dotted lines (a1) and the subdomain organization observed on asection at a medial level of the structure (a2). Color code: yellow, Left-LHB; light purple, internal MHb; blue, Right-LHB; magenta, anterior left external MHb; dark purple, right plus posterior left external MHb; hatched, proliferative pseudo-stratified neuroepithelium. **b-f** Transverse sections after ISH with probes for *ScSox1* (b), *ScPde1a* (c), *ScKctd12b* (d), *ScEnpp2* (e) and *ScProx1* (f), dorsal to the top of each panel. All sections shown were obtained from the same embryo. (b1-f1), (b2-f2), (b3-f3), (b4-f4) and (b5-f5) each show successive adjacent sections. (b1-5), (c1-5), (d1-5), (e1-5) and (f1-5) each show sections from anterior to posterior levels as indicated in (a1). Arrowheads point to lateral habenulae, with black and white arrowheads respectively for the Left- and Right-LHb. Asterisks in (b5) and (f4) show minor *ScSox1* and *ScProx1* posterior expression sites, contra-lateral to their main lateral territory. Dashed lines delimit the subdivision of the medial habenula into external and internal components, as inferred from inner boundaries of the *ScPde1a* and *ScEnpp2* territories. Thin black arrows in (c) and (e) indicate the boundary between the complementary *ScPde1a* and *ScEnpp2* territories within the left external medial habenula. Abbreviations: hc, habenular commissure, pi, pineal; LHb lateral habenula; MHb, medial habenula; ext., external; int., internal, L, left; R, right. Scale bar=100μm.

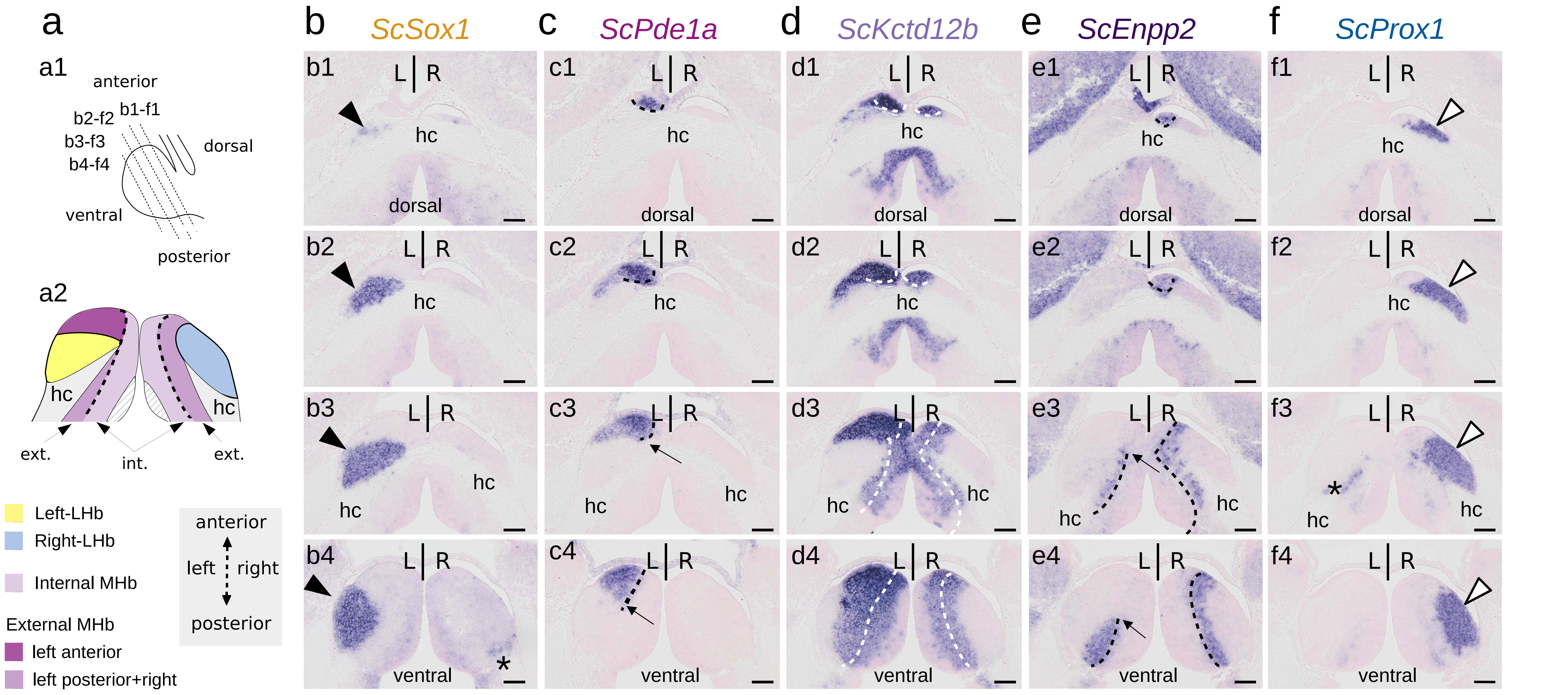

Supplementary Figure 4

See legend on next page

Supplementary Figure 4. Expression of *ScSox1*, *ScPde1a*, *ScKctd12b*, *ScEnpp2* and *ScProx1* along the dorso-ventral axis of stage 31 catshark habenulae. a Schemes showing a left lateral view of stage 31 catshark habenulae, with horizontal section planes indicated by dotted lines (a1) and the subdomain organization observed on a horizontal section at a medial level of the structure (a2). Same color code as in Supplementary Fig.3. **b-f** Horizontal sections after ISH with probes for *ScSox1* (b), *ScPde1a* (c), *ScKctd12b* (d), *ScEnpp2* (e) and *ScProx1* (f), anterior to the top of each panel. All sections were obtained from the same embryo. (b1-f1), (b2-f2), (b3-f3) and (b4-f4) each show successive adjacent sections. (b1-4), (c1-4), (d1-4), (e1-4) and (f1-4) each show sections from dorsal to ventral levels as indicated in (a1). Arrowheads in (b,f) point to the lateral habenulae, with black and white arrowheads respectively for Left- and Right-LHb. Asterisks in (b4) and (f3) show minor *ScSox1* and *ScProx1* posterior expression sites, contra-lateral to their main lateral territory. Dashed lines delimit the subdivision of the medial habenula into external and internal components, as inferred from inner boundaries of the *ScPde1a* and *ScEnpp2* territories. Thin black arrows in (c) and (e) indicate the boundary between the complementary *ScPde1a* and *ScEnpp2* territories within the left external medial habenula. Same abbreviations as in Supplementary Fig.3. Scale bar=100μm.

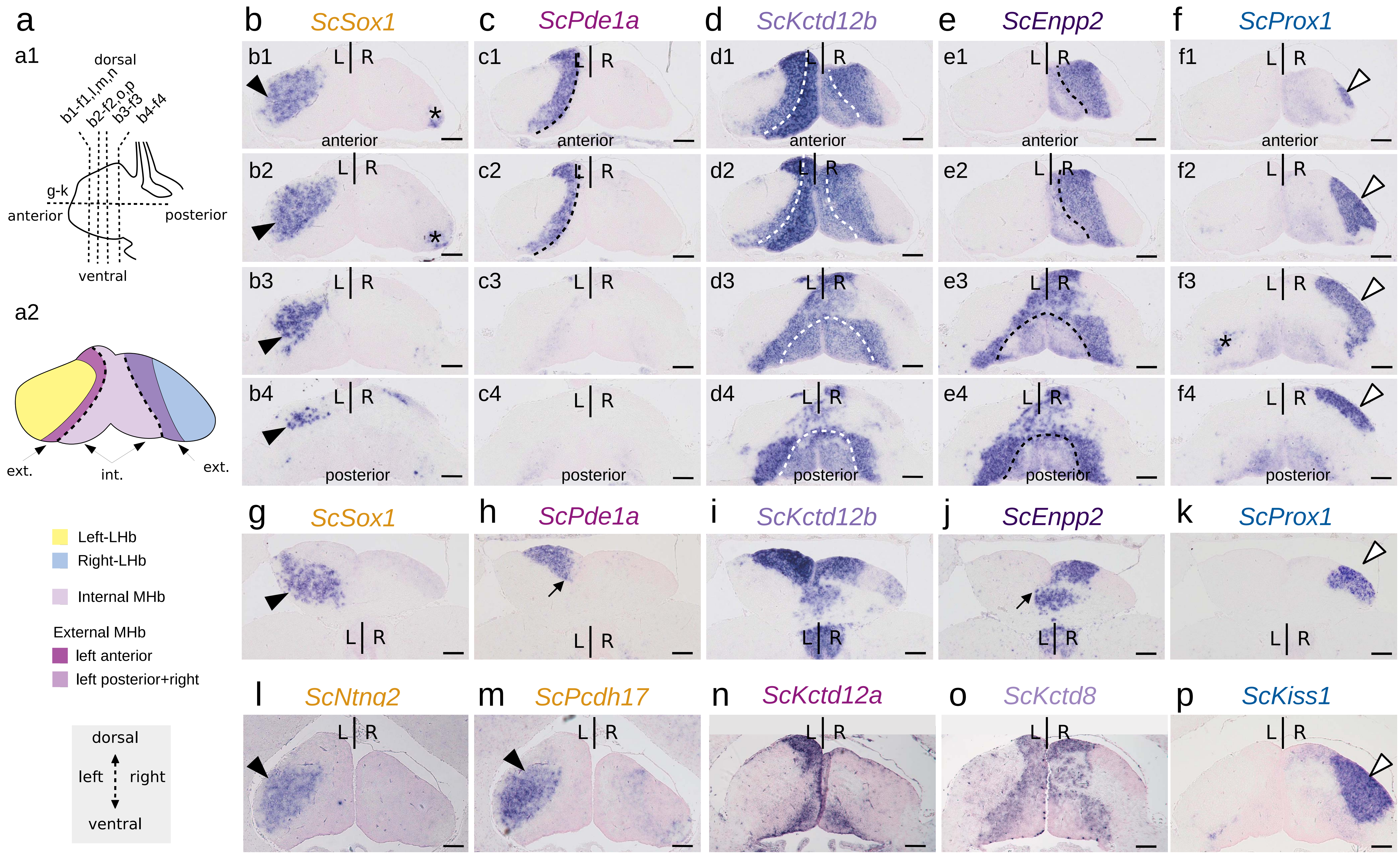

Supplementary Figure 5

See legend on next page

Supplementary Figure 5. Subdomain organization of juvenile catshark habenulae. a Schemes showing a lateral view of juvenile catshark habenulae, with section planes used in (b-p) indicated by dotted lines (a1), and the subdomain organization observed on a transverse section at an anterior level of the structure (a2). Same color code as in Supplementary Fig.3. **b-f** Transverse sections after ISH with probes for *ScSox1* (b), *ScPde1a* (c), *ScKctd12b* (d), *ScEnpp2* (e) and *ScProx1* (f), dorsal to the top of each panel. Sections (b-f) were obtained from the same specimen. (b1-f1), (b2-f2), (b3-f3) and (b4-f4) each show successive adjacent sections. (b1-4), (c1-4), (d1-4), (e1-4), and (f1-4) each show sections from anterior to posterior levels as indicated in (a1). **g-k** Horizontal sections at a medial level of habenulae after ISH with probes for *ScSox1* (g), *ScPde1a* (h), *ScKctd12b* (i), *ScEnpp2* (j) and *ScProx1* (k), anterior to the top of each panel. Sections (g-k) were obtained from the same specimen. (g), (h), (i), (j) and (k) show successive adjacent sections at the levels indicated in (a1). **l-p** Transverse sections after ISH with probes for *ScNtng2* (l), *ScPcdh17* (m), *ScKctd12a* (n), *ScKctd8* (o) and *ScKiss1* (p), dorsal to the top of each panel. Arrowheads point to the lateral habenulae, with black and white arrowheads respectively for Left- and Right-LHb. Asterisks in (b1,b2,f3) show minor *ScSox1* and *ScProx1* posterior expression sites, contra-lateral to their main lateral territory. Dashed lines delimit the subdivision of the medial habenula into external and internal components, as inferred from inner boundaries of the *ScPde1a* and *ScEnpp2* territories. Thin black arrows in (h,j) indicate the boundary between complementary anterior (*ScPde1a*-positive) and posterior (*ScEnpp2*-positive) subdomains of the left external medial habenula. Same abbreviations as in Supplementary Fig.3. Scale bar=100µm.

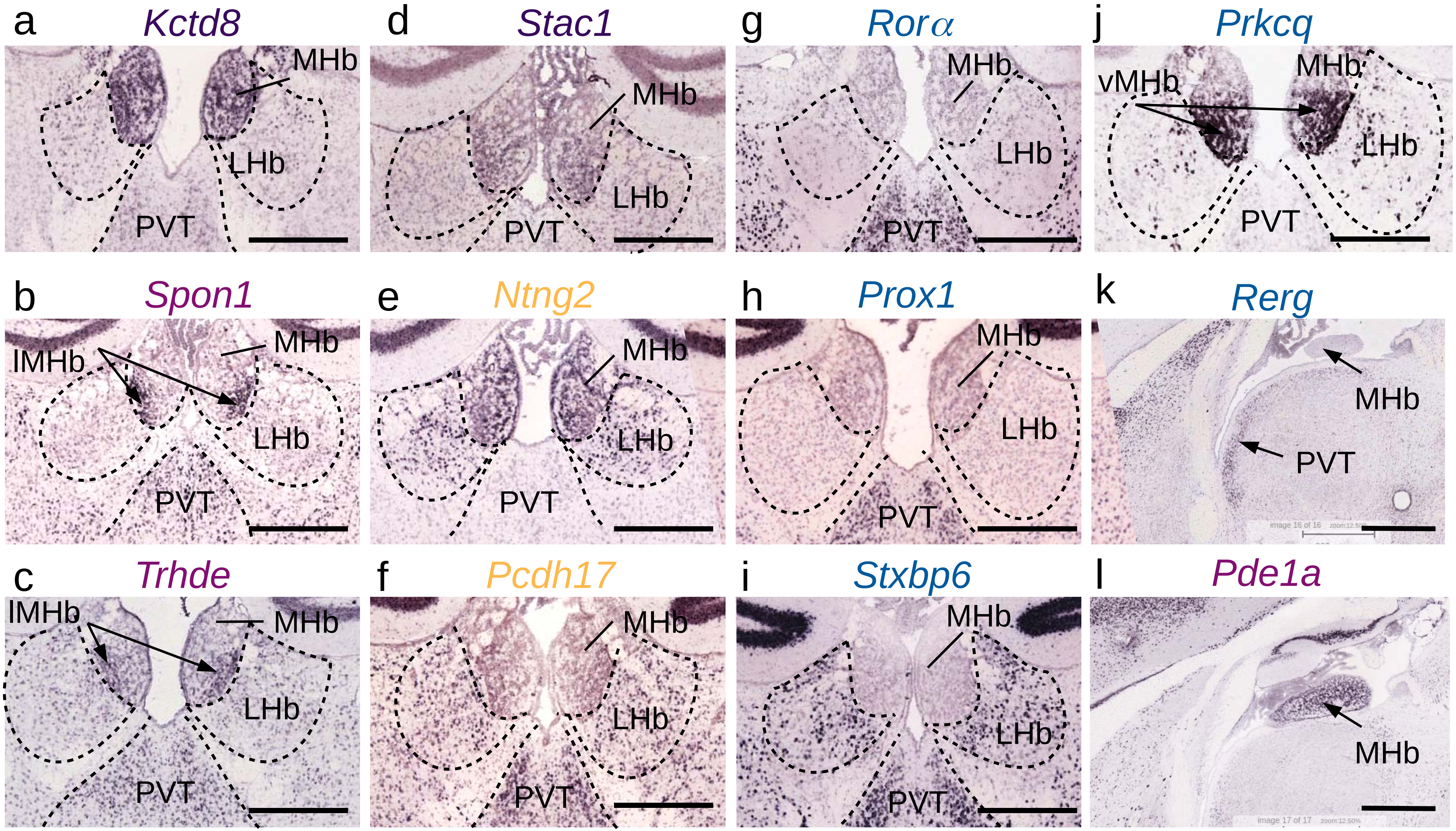

Supplementary Figure 6

See legend on next page

Supplementary Figure 6. Expression of mouse orthologs of catshark Left-LHb, MHb and Right-LHb markers. a-k Images of mouse adult coronal (a-j) or sagittal (k-l) sections following ISH with probes for *Kctd8* (a), *Spon1* (b), *Trhde* (c), *Stac1* (d), *Ntng2* (e), *Pcdh17* (f), *Rorα* (g), *Prox1* (h), *Stxbp6* (i), *Prkcq* (j), *Rerg* (k) and *Pde1a* (l) at the level of the habenulae. The lateral habenulae and the paraventricular nucleus of the thalamus are delimited by dashed lines in (a-j). All images were taken from the Allen Brain Atlas. Gene names are shown with the following color code: orthologs of markers of catshark Left-LHb, yellow; external anterior Left-MHb, magenta; MHb, dark purple; Right-LHb, blue. Abbreviations: LHb, lateral habenula; lMHb, lateral medial habenula; vMHb, ventral medial habenula; MHb, medial habenula; PVT, paraventricular nucleus of the thalamus. Scale bar=420μm in (a-j) and 840μm in (k-l).

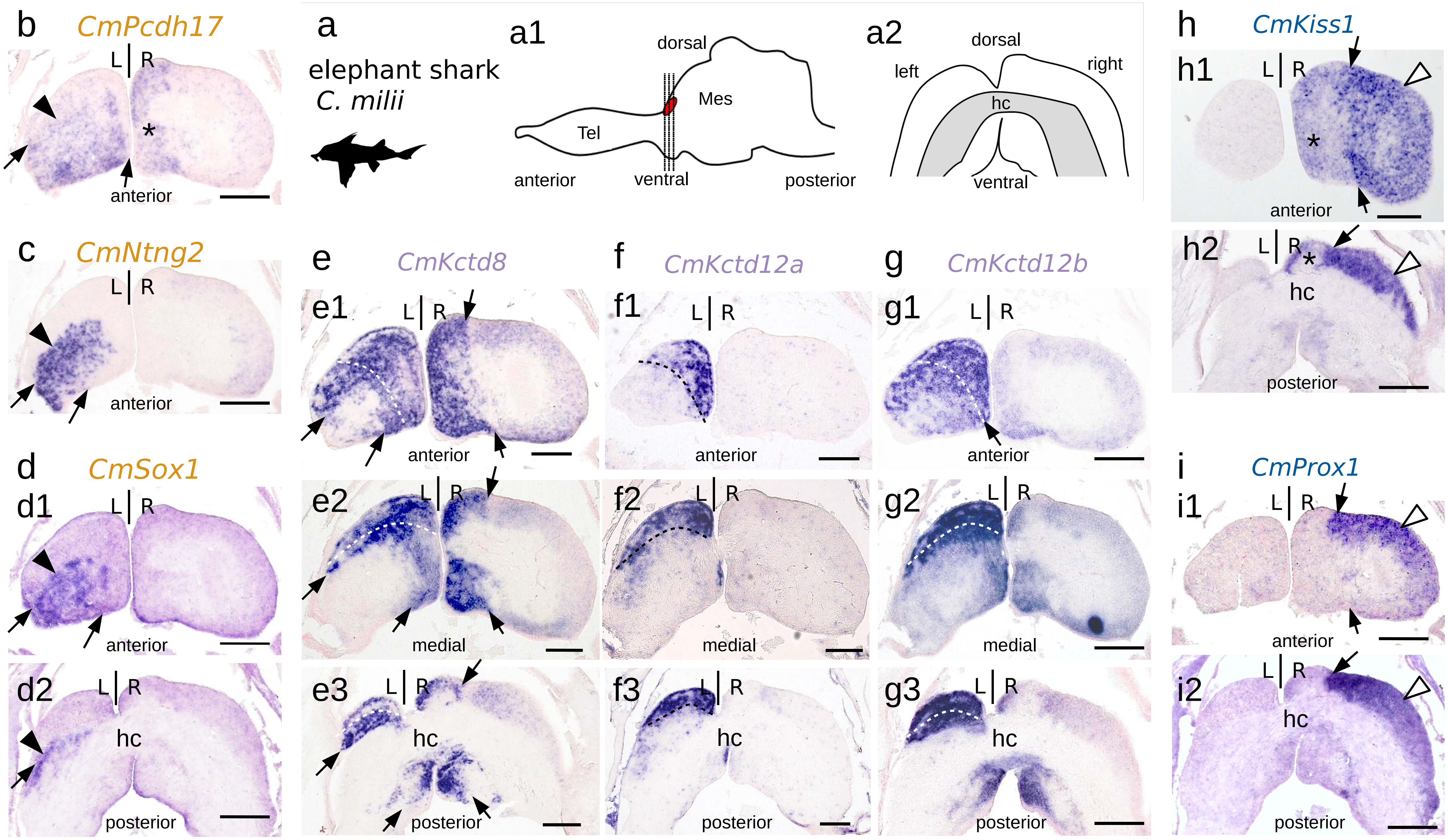

Supplementary Figure 7

See legend on next page

Supplementary Figure 7. Subdomain organization of habenulae in the elephant shark *Callorhinchus milii*. a Schemes showing a left lateral view of the elephant shark brain, with the location of the habenula in red and dotted lines indicating transverse section planes at anterior, medial and posterior levels (a1) and a section at a posterior organ level with the habenular commissure in gray (a2). **b-i** Transverse sections of elephant shark embryonic habenulae (stage 36) after ISH with probes for *CmPcdh17* (b), *CmNtng2* (c), *CmSox1* (d), *CmKctd8* (e), *CmKctd12a* (f), *CmKctd12b* (g), *CmKiss1* (h), and *CmProx1* (i), dorsal to the top. All sections were obtained from the same specimen. The level of the sections along the antero-posterior axis is indicated on each panel. Black and white arrowheads point to lateral territories respectively co-expressing *CmPcdh17*/*CmNtng2*/*CmSox1* on the left, and *CmKiss1/CmProx1* on the right. Thin arrows indicate their boundary with *CmKctd8/12a/12b*-positive, medial habenula territories. Asterisks in (b,h1,h2) indicate medial territories where *CmPcdh17* (b) and *CmKiss1* (h1,h2) are expressed in addition to their major lateral territories. Dotted lines in (e,f,g) delimit two subdomains within the left medial habenula, respectively positive or negative for *Kctd12a*. Abbreviations: hc, habenular commissure; Mes, mesencephalon; Tel, telencephalon; L, left; R, right. Scale bar=500μm.

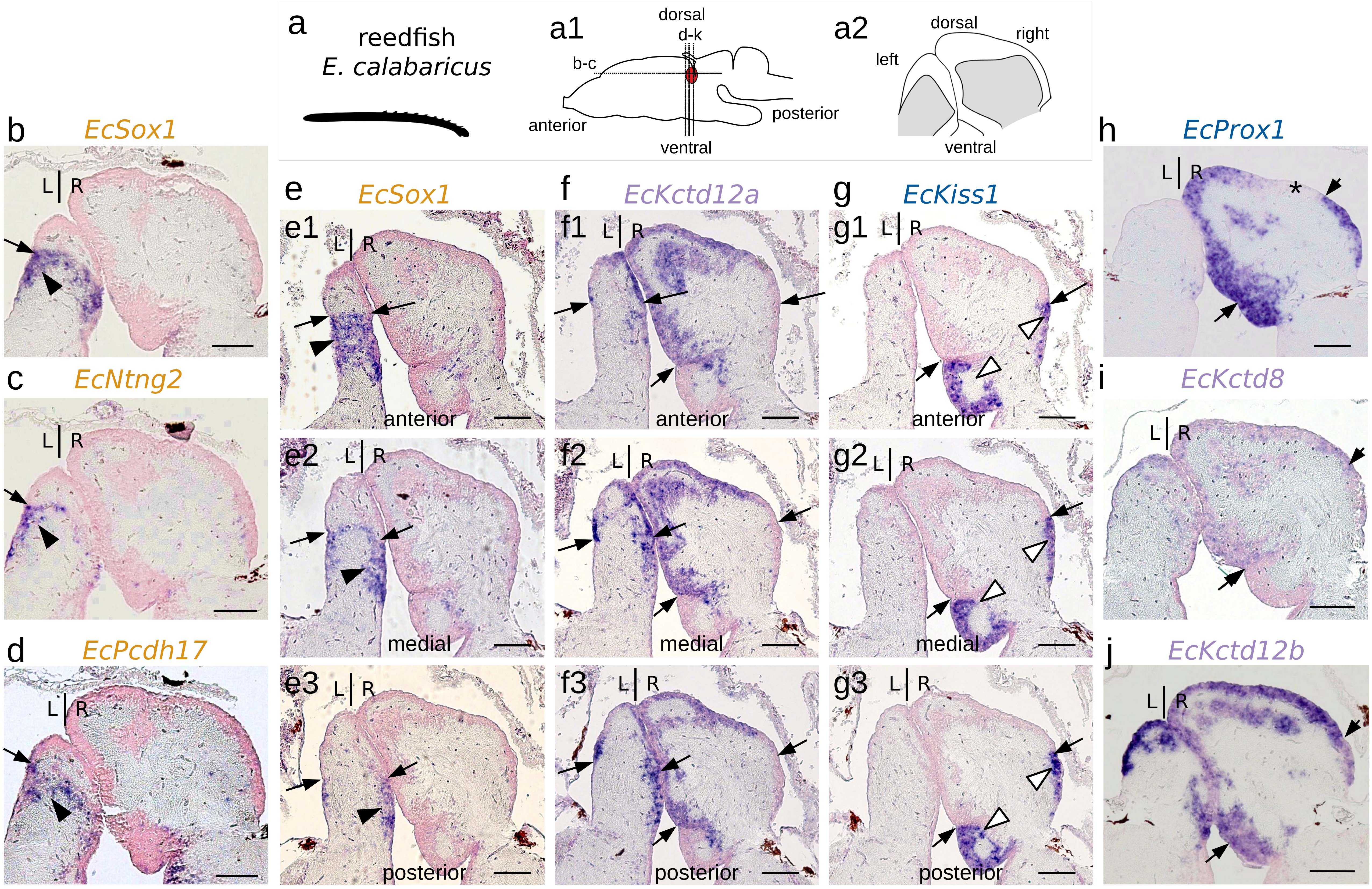

Supplementary Figure 8

See legend on next page

Supplementary Figure 8. Subdomain organization of habenulae in the reedfish *Erpetoichthys calabaricus*. a Schemes showing a left lateral view of the reedfish brain, with the location of the habenula in red and dotted lines indicating section planes at anterior, medial and posterior levels (a1), and a section at a medial organ level with tract zones in gray (a2). **b-j** Transverse sections of reedfish juvenile habenulae after ISH with probes for *EcSox1* (b,e), *EcNtng2* (c), *EcPcdh17* (d), *EcKctd12a* (f), *EcKiss1* (g), *EcProx1* (h), *EcKctd8* (i) and *EcKctd12b* (j), dorsal to the top. Sections (b-d) were obtained from the same specimen, same for sections (e-g) and sections (h-j). (e1,f1,g1), (e2,f2,g2) and (e3,f3,g3) respectively show sections at anterior, medial and posterior habenula levels. Black and white arrowheads point to ventral territories respectively co-expressing *EcSox1/EcNtng2/EcPcdh17* on the left and expressing *EcKiss1* on the right. Thin arrows indicate their boundary with *EcKctd8/12a*-positive dorsal habenula territories. *EcProx1* expression is restricted to the right side, but spans both dorsal and ventral subdomains, except for a minor dorsal territory, labeled by an asterisk (h). Abbreviations: L, left; R, right. Scale bar=100µm.

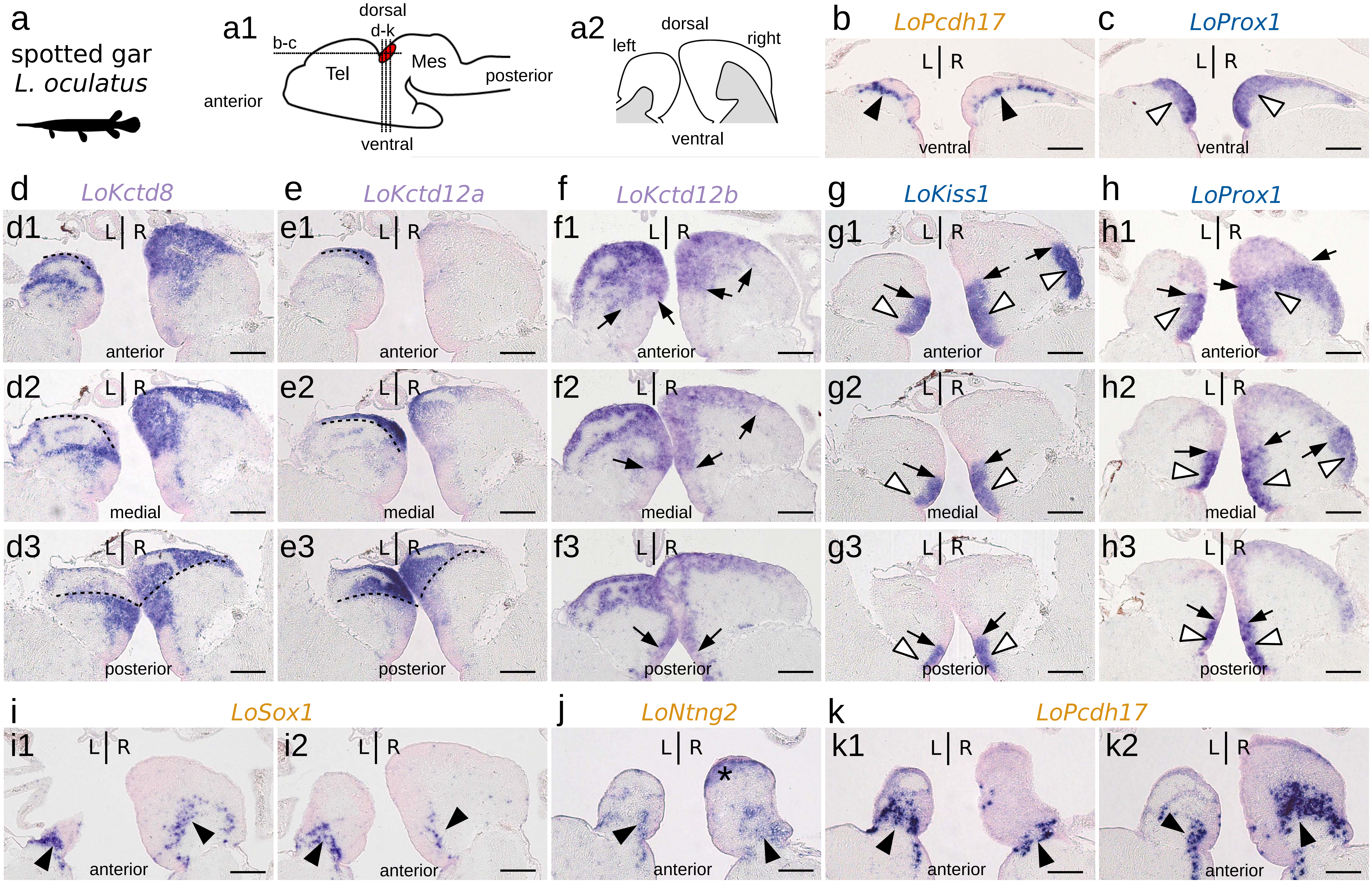

Supplementary Figure 9

See legend on next page

Supplementary Figure 9. Subdomain organization of habenulae in the spotted gar *Lepisosteus oculatus*. a Schemes showing a left lateral view of the spotted gar brain, with the location of the habenula in red and dotted lines indicating section planes at anterior, medial and posterior levels (a1), and a transverse section at a medial organ level with tract zones in gray (a2) . **b-c** Adjacent horizontal sections of the habenulae of a same spotted gar juvenile after ISH with probes for *LoPcdh17* (b) and *LoProx1* (c), anterior to the top. **d-k** Transverse sections after ISH with probes for *LoKctd8* (d), *LoKctd12a* (e), *LoKctd12b* (f), *LoKiss1* (g), *LoProx1* (h), *LoSox1* (i), *LoNtng2* (j), and *LoPcdh17* (k), dorsal to the top. Sections (d,e,g) were obtained from the same specimen, same for sections (j,k). (b,c), (d1,e1,f1,g1,h1,i1,i2,j,k1,k2), (d2,e2,f2,g2,h2) and (d3,e3,f3,g3,h3) respectively show sections at ventral, anterior, medial and posterior habenula levels. Black and white arrowheads point to bilateral ventral territories respectively co-expressing *LoSox1/LoNtng2/LoPcdh17* and *LoKiss1/LoProx1*. Thin arrows indicate their boundary with *LoKctd8/12b*-positive dorsal habenula territories. *LoNtng2* expression includes a dorsal territory, labeled by an asterisk in (j), in addition to its ventral one. Dotted lines in (d,e) delimit two subdomains, respectively positive or negative for *Kctd12a*, within the dorsal habenulae. Abbreviations: Mes, mesencephalon; Tel, telencephalon; L, left; R, right. Scale bar=100µm.

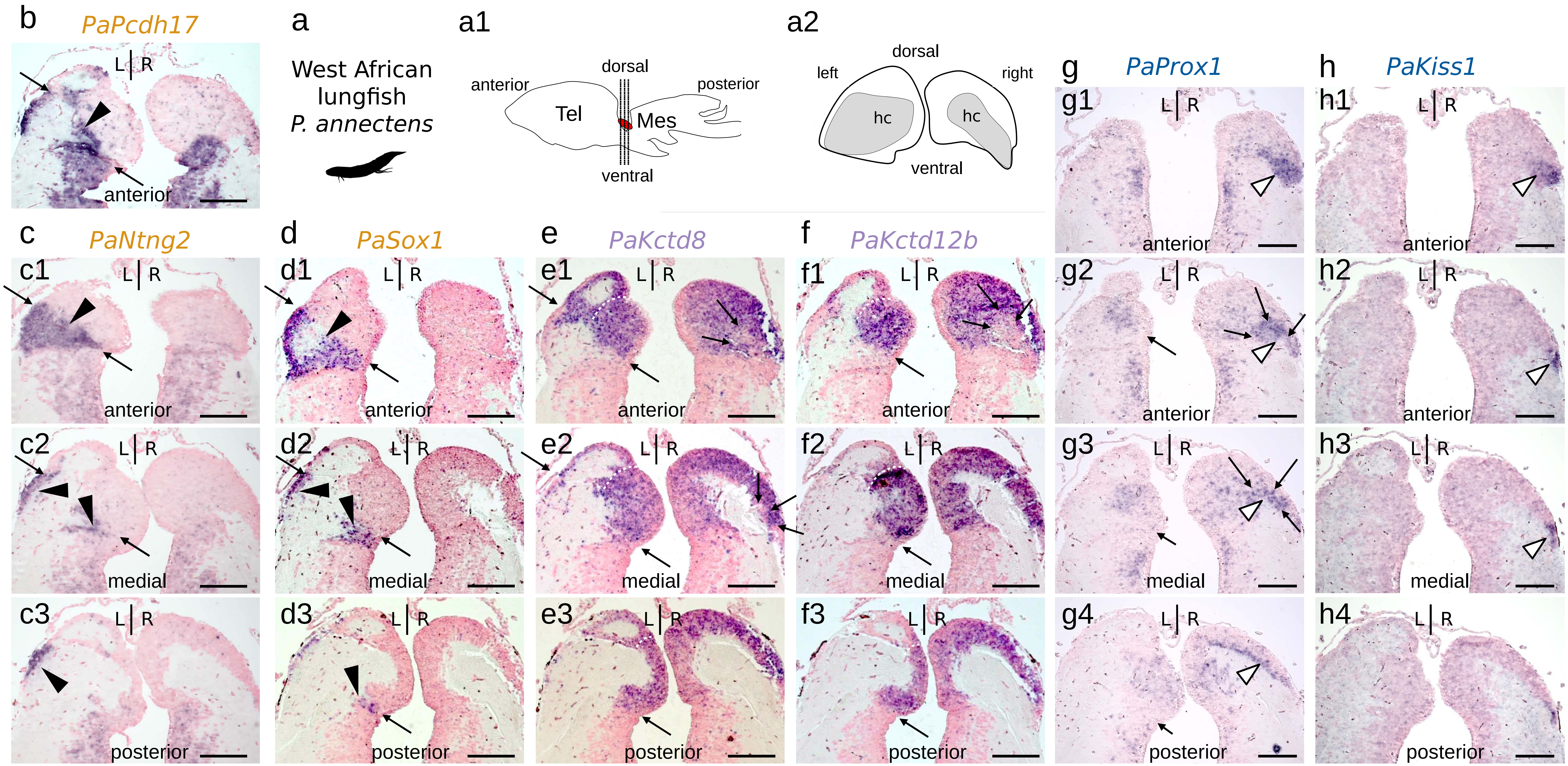

Supplementary Figure 10

See legend on next page

Supplementary Figure 10. Subdomain organization of habenulae in the lungfish *Protopterus annectens*. a Schemes showing a left lateral view of the lungfish brain, with the location of the habenula in red and dotted lines indicating section planes at anterior, medial and posterior levels (a1), and a section at a medial organ level with tract zones in gray (a2). **b-h** Transverse sections of habenulae from lungfish juveniles, after ISH with probes for *PaPcdh17* (b), *PaNtng2* (c), *PaSox1* (d), *PaKctd8* (e), *PaKctd12b* (f), *PaProx1* (g) and *PaKiss1* (h), dorsal to the top. (c1,d1,e1,f1,g1, g2,h1,h2), (c2,d2,e2,f2,g3,h3) and (c3,d3,e3,f3,g4,h4) respectively show sections at anterior, medial and posterior habenula levels. Black arrowheads point to left-restricted *PaSox1*. White arrowheads point to right-restricted *PaProx1* and *PaKiss1* lateral territories. Thin arrows indicate the boundaries of the *PaSox1* and *PaProx1* territories with the *PaKctd8*-positive medial territory, on the left and the right sides respectively. White dotted lines in (d,e) delimit two subdomains, respectively positive or negative for *Kctd12b*, within the *PaKctd8*-positive medial habenula. Abbreviations: hc, habenular commissure; Mes, mesencephalon; Tel, telencephalon; L, left; R, right. Scale bar=250μm.

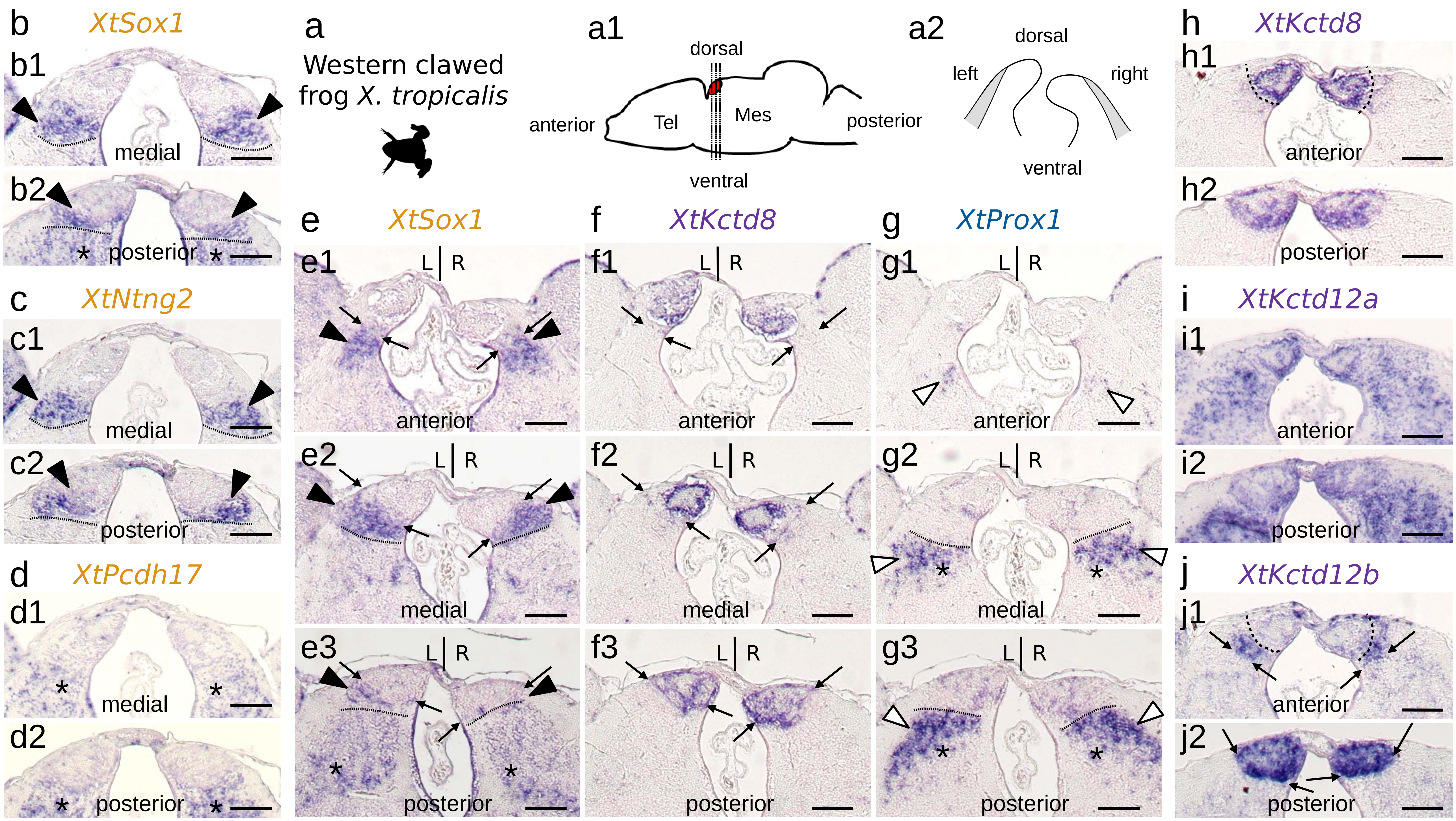

Supplementary Figure 11

See legend on next page

Supplementary Figure 11. Subdomain organization of habenulae in the Western clawed frog *Xenopus tropicalis.* a Schemes showing a left lateral view of the frog brain, with the location of habenula in red and dotted lines indicating section planes at anterior, medial and posterior levels (a1), and a section at a medial organ level with tract zones in gray (a2). **b-j** Transverse sections of the habenulae of a frog juvenile after ISH with probes for *XtSox1* (b,e), *XtNtng2* (c), *XtPcdh17* (d), *XtKctd8* (f,h), *XtKctd12a* (i), *XtKctd12b* (j), and *XtProx1* (g), dorsal to the top. Sections (b,c,d) were obtained from the same specimen, same for sections (e,f,g) and sections (h,i,j). (e1,f1,g1,h1,i1,j1), (b1,c1,d1,e2,f2,g2) and (b2,c2,d2,e3,f3,g3,h2,i2,j2) respectively show sections at anterior, medial and posterior habenula levels. Black and white arrowheads respectively point to a bilateral lateral habenula territory co-expressing *XtSox1*/*XtNtng2* (but not *XtPcdh17*), and an adjacent one expressing *XtProx1*, located more ventrally and excluded from anterior-most levels. Thin arrows indicate the medial/dorsal boundary of the *XtSox1/XtNtng2* territory with medial *XtKctd8/12b*-positive habenula territories. In addition to its habenular territory, *XtSox1* expression expands into a *XtPcdh17/XtProx1*-positive thalamic territory labeled by an asterisk at medial to posterior levels (b2,d1,d2,e3,g2,g3). Dotted lines in (b,c,e,g) delineate the boundary between the *XtSox1/XtNtng2* and the *XtProx1* territories. Dashed lines in (h1,j1) delimit two anterior subdomains, respectively expressing *XtKctd8* and *XtKctd12b*, within the medial habenulae. Abbreviations: Mes, mesencephalon; Tel, telencephalon; L, left; R, right. Scale bar=50μm.

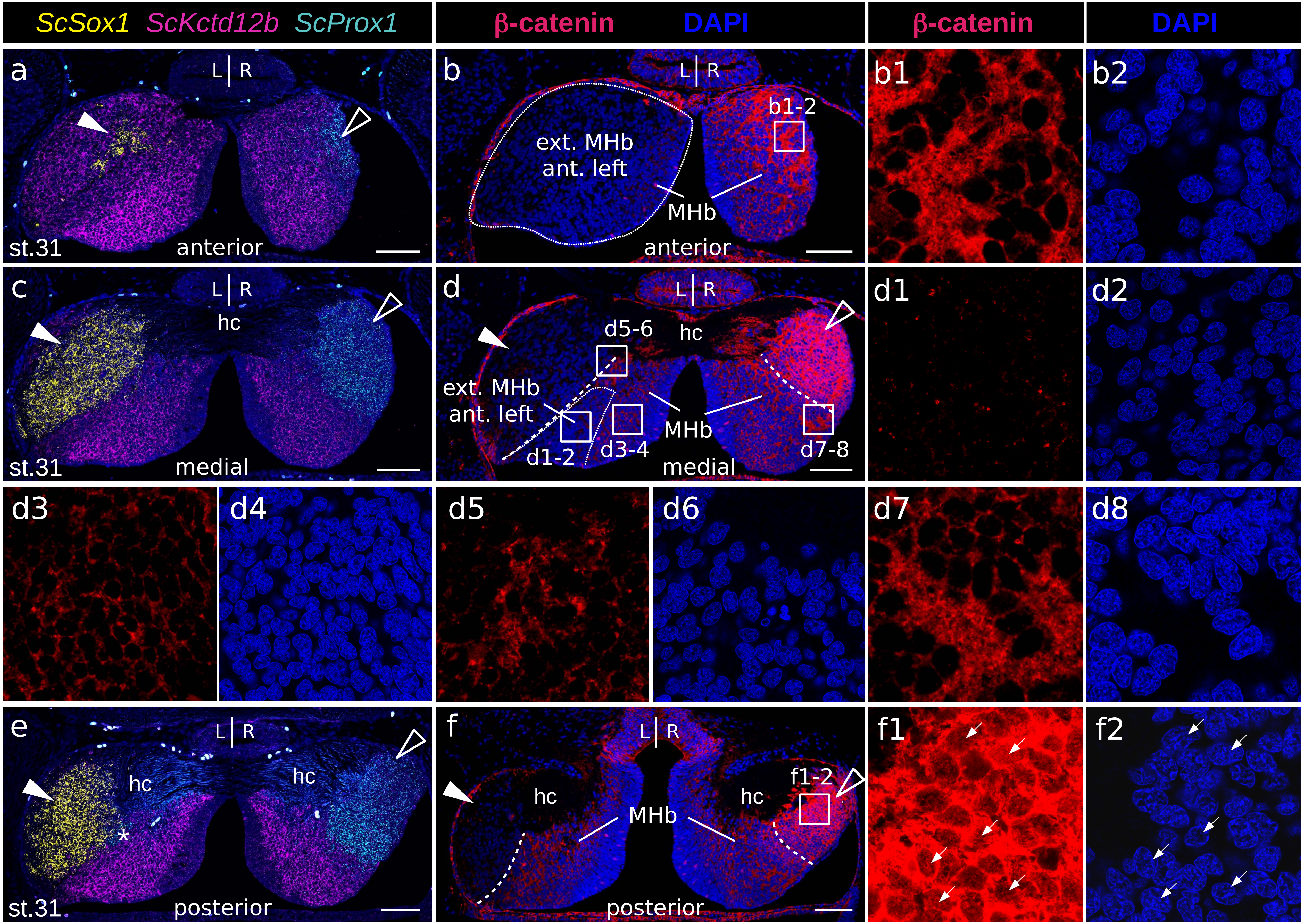

Supplementary Figure 12

See legend on next page

Supplementary Figure 12. Heterogeneity of β-catenin distribution in the catshark medial habenula at stage 31. a-f Confocal images of transverse sections of developing catshark habenulae (stage 31) after HCR-FISH (hybridization chain-reaction based fluorescent *in situ* hybridization) with signals for *ScSox1*, *ScKctd12b* and *ScProx1* in respectively yellow, magenta, and cyan (a,c,e), and after IHC using an antibody directed against β-catenin (red) (b,d,f). DAPI-stained nuclei are shown in blue. All sections were obtained from the same embryo. (a,b), (c,d) and (e,f) each show adjacent sections, at anterior, medial and posterior levels, respectively. (b1,b2), (d1-8) and (f1,f2) show magnifications of the territories boxed in (b), (d) and (f), with β-catenin signals in (b1,d1,d3,d5,d7,f1) and DAPI signals in (b2,d2,d4,d6,d8,f2). White and empty arrowheads in (a,c,e) respectively point to Left- and Right-LHb, an asterisk in (e) marks a minor *ScProx1* territory on the left. Thin white arrows in (f1,f2) point to β-catenin-labeled nuclei. A dashed line in (d,f) indicates the approximate boundary between lateral and medial habenula territories. A dotted line in (b,d) delimits the left-restricted *ScPde1a*-expressing subdomain, located anteriorly, in the external part of the MHb. A strong nuclear β-catenin signal is observed in the right lateral habenula (f1,f2), but not in its left counterpart as described in Fig.3. In the medial habenula, no nuclear β-catenin signal could be observed, whatever the level (external or internal, anterior or posterior) and the side (b,d,f,b1,b2,d1-8). However, β-catenin expression is heterogeneous in the medial habenula, with cytoplasmic signals present on both sides and all levels (b1,b2,d3-8), except in a left-restricted subdomain, located at anterior levels of the external part of the medial habenula (b,d1,d2). Based on its location, this territory corresponds to the left-restricted *ScPde1a*-expressing MHb subdomain. Abbreviations: ant., anterior; ext., external; hc, habenular commissure; MHb, medial habenula; L, left; R, right; st., stage. Scale bars=100μm.

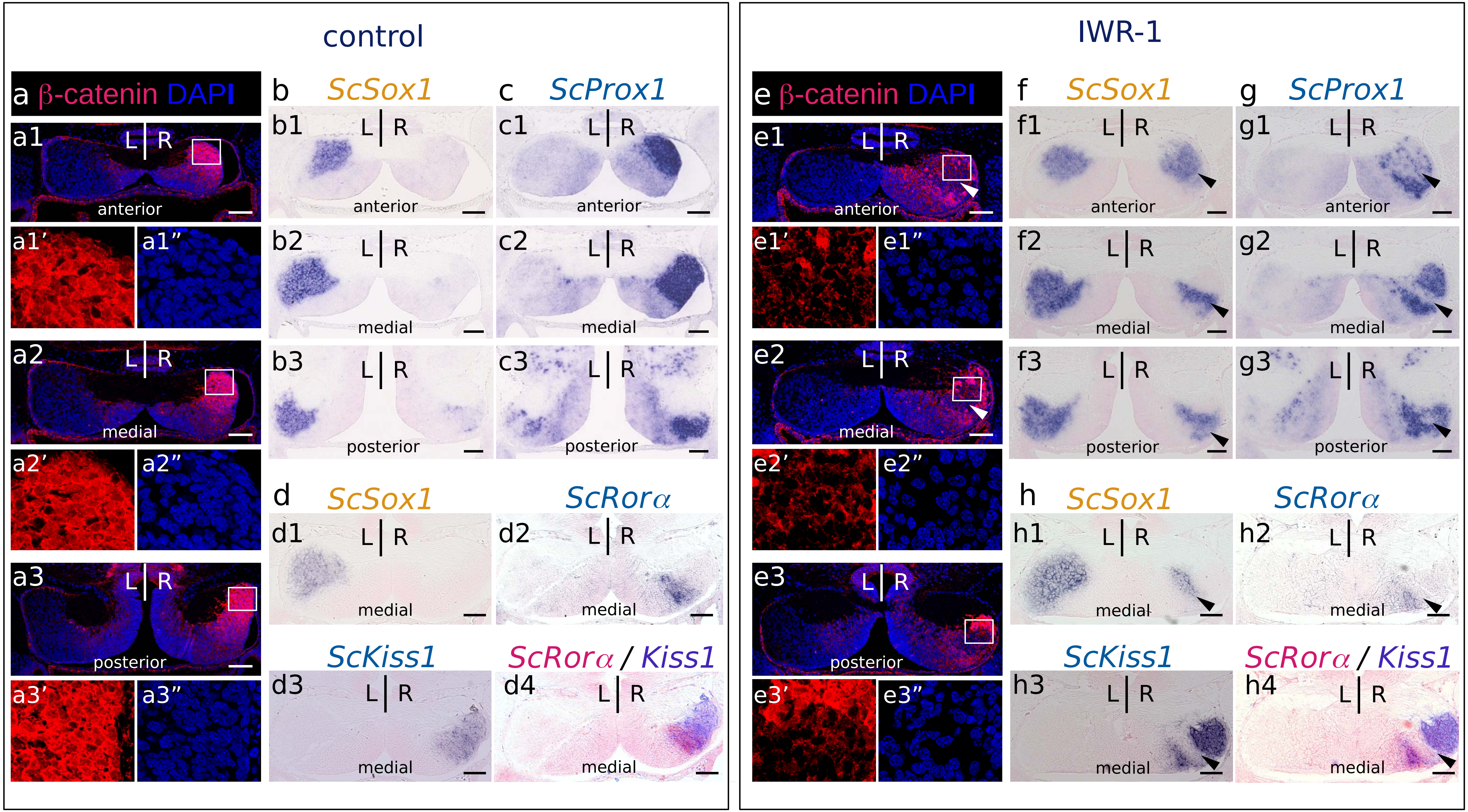

Supplementary Figure 13

See legend on next page

Supplementary Figure 13. Phenotypes observed along the antero-posterior axis of catshark stage 31 habenulae following IWR-1 treatment. a-l Transverse sections of catshark stage 31 habenulae from control (a-d) and IWR-1-treated (e-h) embryos, after IHC with an antibody directed against β-catenin, with β-catenin in red and DAPI-stained nuclei in blue (a,e), and after ISH with probes for *ScSox1* (b,f,d1,h1), *ScProx1* (c,g), *ScRorα* (d2,h2), *ScKiss1* (d3,h3). (d4,h4) show the superimposition of *ScKiss1* (false color, purple) and *ScRora* (false color, magenta) signals, as inferred from ISH on adjacent sections. Section levels along the antero-posterior axis are indicated at the bottom of each panel (a1-c1,e1-g1, anterior; a2-c2,e2-g2,d1-3,h1-3, medial; a3-c3,e3-g3, posterior), dorsal is to the top. (b,c) were obtained from the same embryo, same for (f,g), (d1-d4) and (h1-h4). (b1,c1) are adjacent sections, same for (b2,c2), (b3,c3), (f1,g1), (f2,g2), (f3,g3), (d1,d2,d3) and (h1,h2,h3). (a1',a1"), (a2',a2"), (a3',a3"), (e1',e1"), (e2',e2"), and (e3',e3") show magnifications of the territories boxed in (a1), (a2), (a3), (e1), (e2) and (e3), respectively, with β-catenin signals in (a1',a2',a3',e1',e2',e3') and DAPI signals in (a1",a2",a3",e1"e2",e3"). Arrowheads in (e-h) show a loss of nuclear β-catenin accumulation in the lateral right habenula (e) and an expansion of *ScSox1* expression to this territory (f,h1), concomitant with a loss of *ScProx1* (g), *ScKiss1* (h3,h4) and *ScRorα* (h2,h4) expression. Abbreviations: L, left; R, right. Scale bar=100μm.

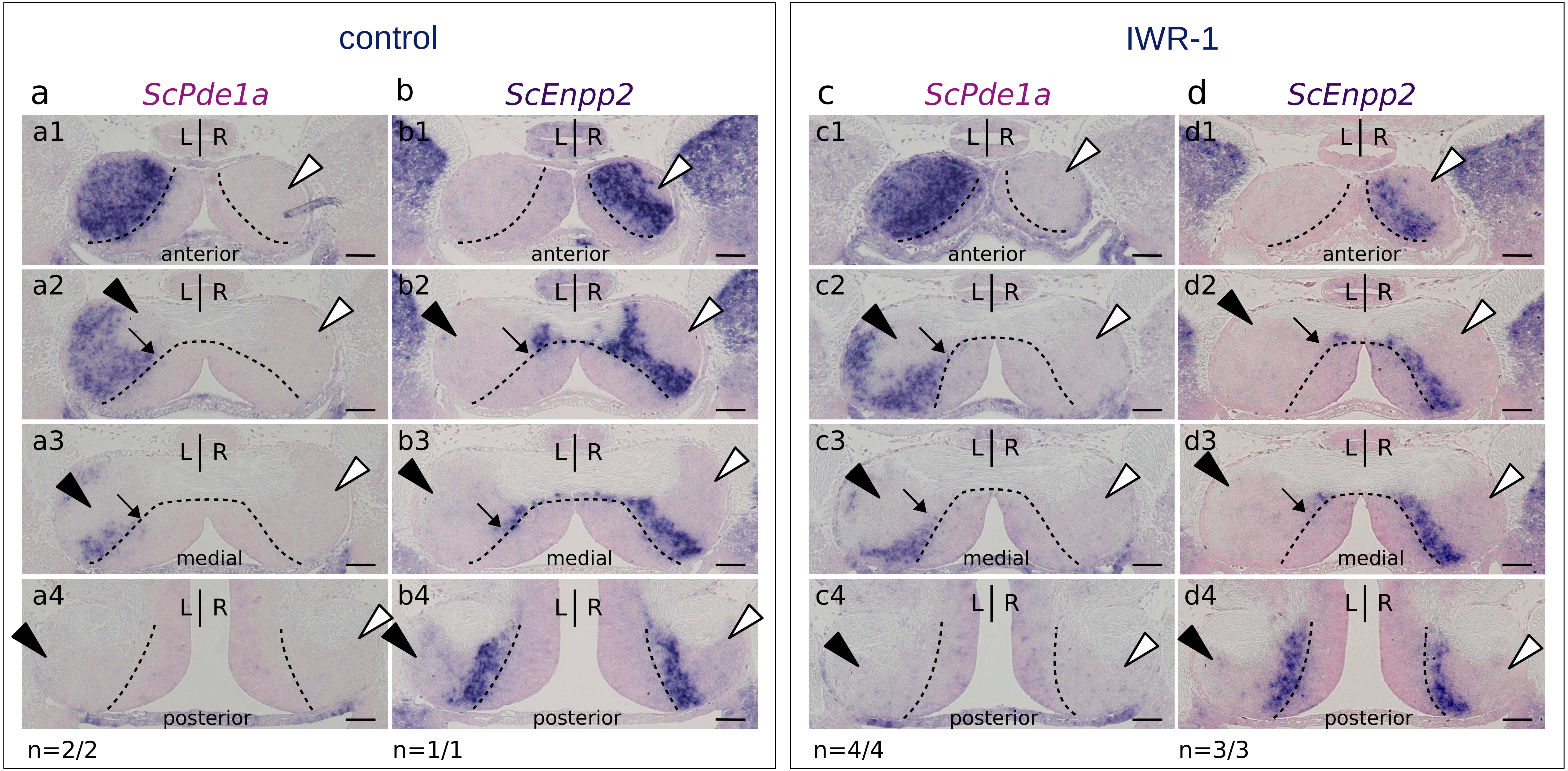

Supplementary Figure 14

See legend on next page

Supplementary Figure 14. IWR-1 treatment at stage 29 has no effect on *ScPde1a* and *ScEnpp2* expression in developing catshark habenulae. a-d Transverse sections of catshark stage 31 habenulae from control (a,b) and IWR-1 treated (c,d) embryos, after ISH with probes for *ScPde1a* (a,c) and *ScEnpp2* (b,d). Sections (a1-4), (b1-4), (c1-4) and (d1-4) progress along the antero-posterior axis of the habenulae from anterior to posterior levels, dorsal is to the top. (a,b) are adjacent sections from the same embryo, same for (c,d). (a1,b1) are adjacent sections, same for (a2,b2), (a3,b3), (a4,b4), (c1,d1), (c2,d2), (c3,d3) and (c4,d4). Dashed lines delimit internal and external components of the medial habenula, a thin arrow points to the boundary between the complementary *ScPde1a* and *ScEnpp2* territories in the left external medial habenula. Black and white arrowheads show the location of left and right lateral habenulae, respectively. "n=" refers to the number of embryos with the same asymmetry pattern for the makers shown over the total number of embryos analyzed. Abbreviations: L, left; R, right. Scale bar=100μm.

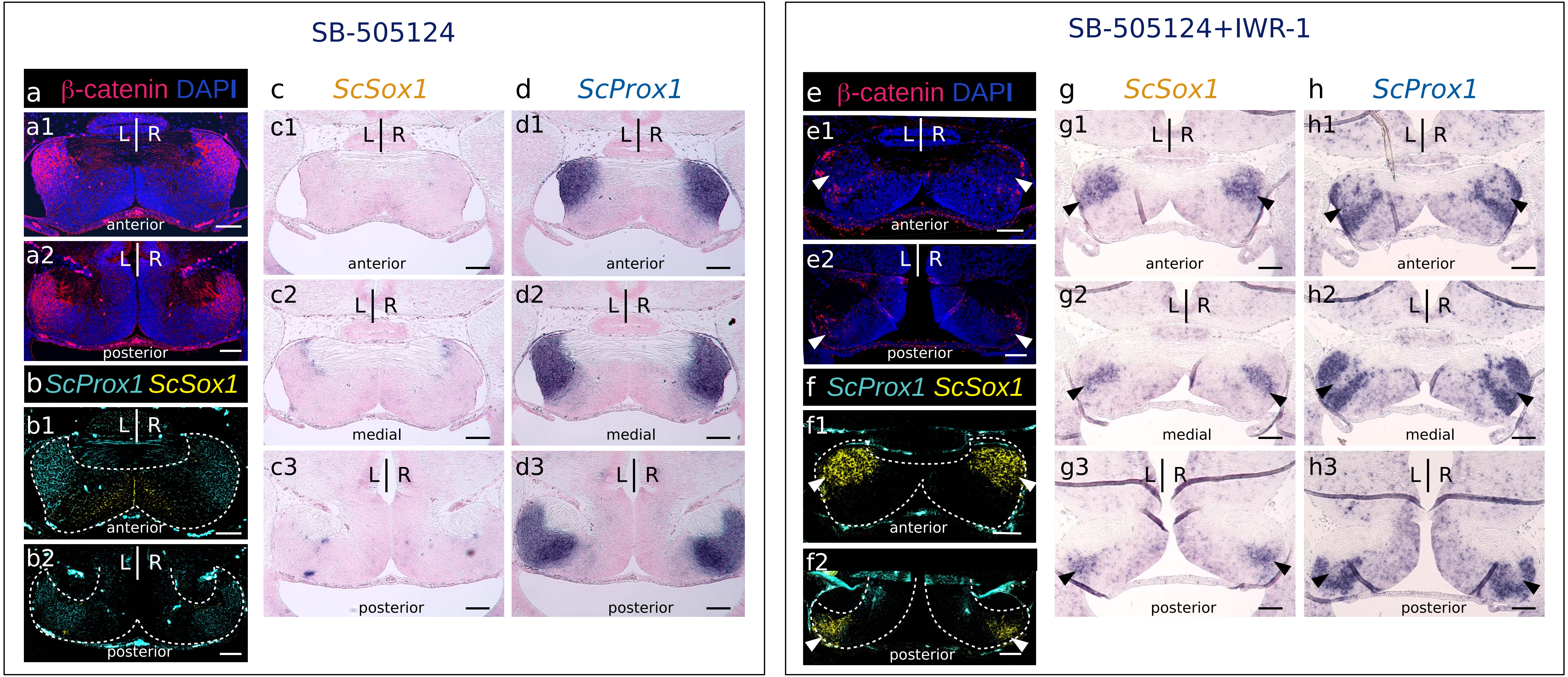

Supplementary Figure 15

See legend on next page

Supplementary Figure 15. Phenotypes observed along the antero-posterior axis of catshark stage 31 habenulae following double SB-505124 and IWR-1 treatment. a-h Transverse sections of catshark stage 31 habenulae from SB-505124 (a-d) and double SB-505124-+IWR-1-treated (e-h) embryos, after IHC with an antibody directed against β-catenin, with β-catenin in magenta and DAPI-stained nuclei in blue (a,e), HCR-FISH with territories of *ScSox1* and *ScProx1* in yellow and cyan, respectively (b,f), and ISH with probes for *ScSox1* (c,g) and *ScProx1* (d,h). Section levels along the antero-posterior axis are indicated at the bottom of each panel (a1-h1, anterior; c2,d2,g2,h2, medial; a2,b2,c3,d3,e2,f2,g3,h3, posterior), dorsal to the top. (a,b) were obtained from the same embryo, same for (c,d), (e,f) and (g,h). (c1,d1) are adjacent sections, same for (c2,d2), (c3,d3), (g1,h1), (g2,h2) and (g3,h3). A right isomerism is observed at all axial levels in SB-505124-treated embryos (a-d). Arrowheads in (e-h) point to bilateral territories where nuclear β-catenin accumulation (e) and *ScProx1* expression (f,h) are lost, and where *ScSox1* expression is rescued in lateral habenulae of double SB-505124-+IWR-1-treated embryos. These territories are never observed in embryos only treated with SB-505124 (a-d). Abbreviations: L, left; R, right. Scale bar=100μm.

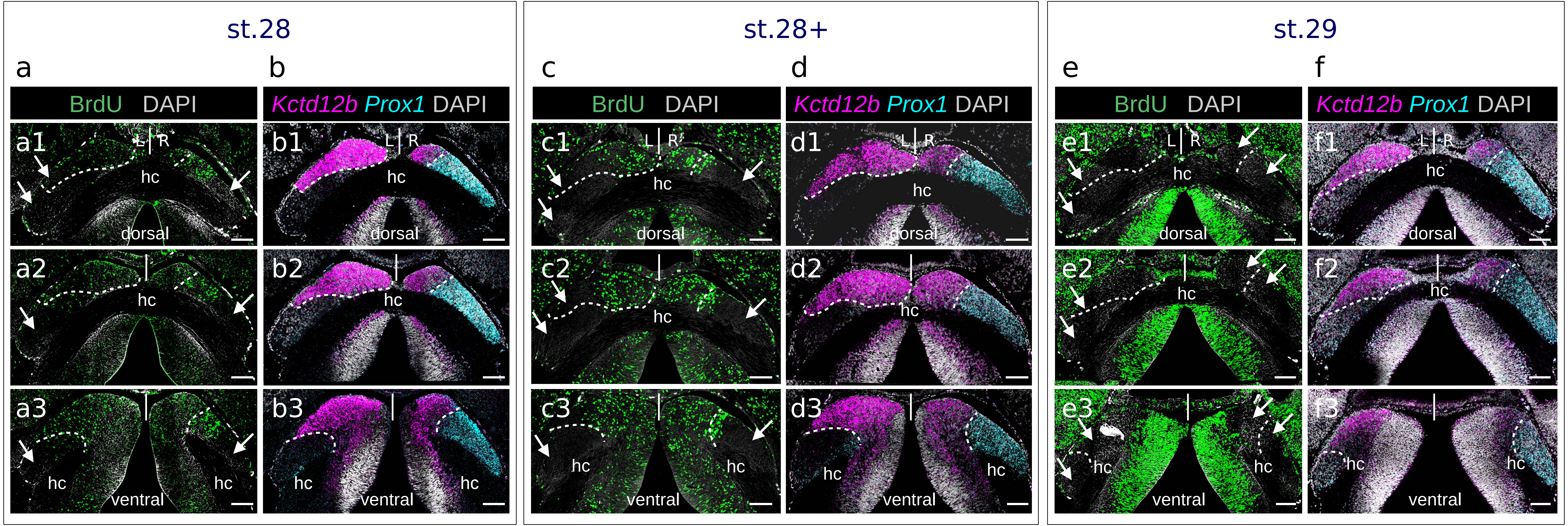

Supplementary Figure 16

See legend on next page

Supplementary Figure 16. Spatial and temporal regulation of progenitor cell cycle exits in developing catshark habenulae. a-f Horizontal sections of catshark stage 31 habenulae following exposure of embryos to BrdU pulses at stage 28 (a,b), 28+ (c,d), and 29 (e,f), anterior to the top. (a,c,e) show confocal images following IHC using an antibody directed against BrdU (green). DAPI-stained nuclei are shown in in gray. (b,d,f) respectively show confocal images after double ISH with probes for *ScKctd12b* (magenta) and *ScProx1* (cyan), with DAPI-stained nuclei in gray. (a1,b1), (a2,b2) and (a3,b3) each show adjacent sections of the same embryo from dorsal to ventral levels, respectively, same for (c1,d1), (c2,d2), (c3,d3), (e1,f1), (e2,f2) and (e3,f3). White dashed lines in (b,d,f) delimit the border between the medial (MHb) and lateral (LHb) habenulae, as inferred from *ScKctd12b* expression. The approximate location of this border is also shown on adjacent BrdU-labeled sections (a,c,e). Thin arrows point to BrdU-negative territories in the differentiating habenulae. Abbreviations: hc, habenular commissure; L, left; R, right; st., stage. Scale bar=100μm.

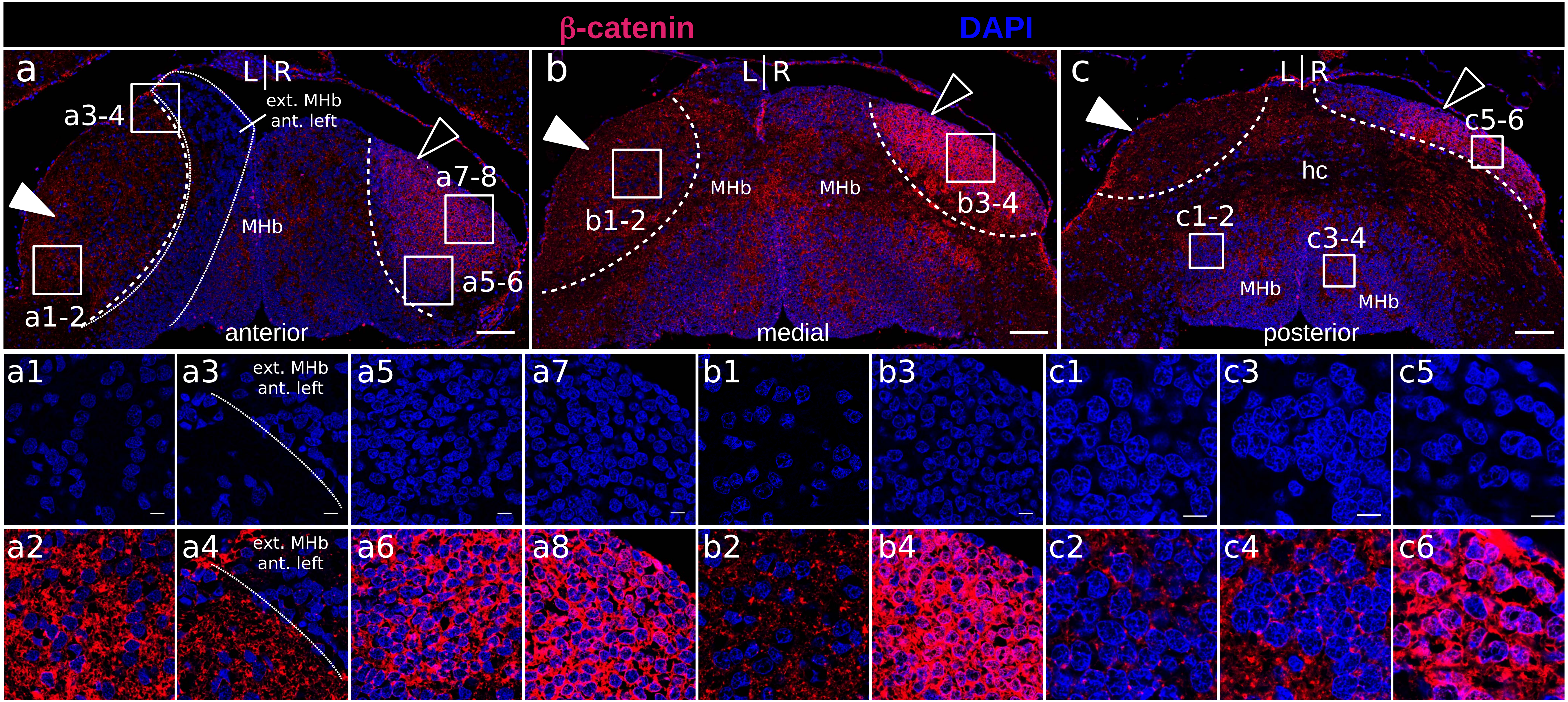

Supplementary Figure 17

See legend on next page

Supplementary Figure 17. Asymmetric β-catenin signals persist in juvenile catshark habenulae. a-c Confocal images of transverse sections of catshark juvenile habenulae after IHC using an antibody directed against β-catenin, dorsal to the top. Sections in (a), (b), and (c) are shown at anterior, medial, and posterior levels, respectively. DAPI-stained nuclei are shown in blue. All sections were obtained from the same embryo. (a1-8), (b1-4) and (c1-6) show magnifications of the territories boxed in (a), (b) and (c), with DAPI signals in (a1,a3,a5,a7,b1,b3,c1,c3,c5) and DAPI/ β-catenin signals in (a2,a4,a6,a8,b2,b4,c2,c4,c6). White arrowheads and black arrowheads point to Left- and Right-LHb, respectively. Dotted lines delineate the boundary between lateral and medial habenulae. Dashed lines delimit the left-restricted anterior subterritory (*ScPde1a*-positive) of the external medial habenula. Abbreviations: hc, habenular commissure; L, left; R, right. Scale bar=100μm.

a *Pcdh10/10l/17/18/19* family

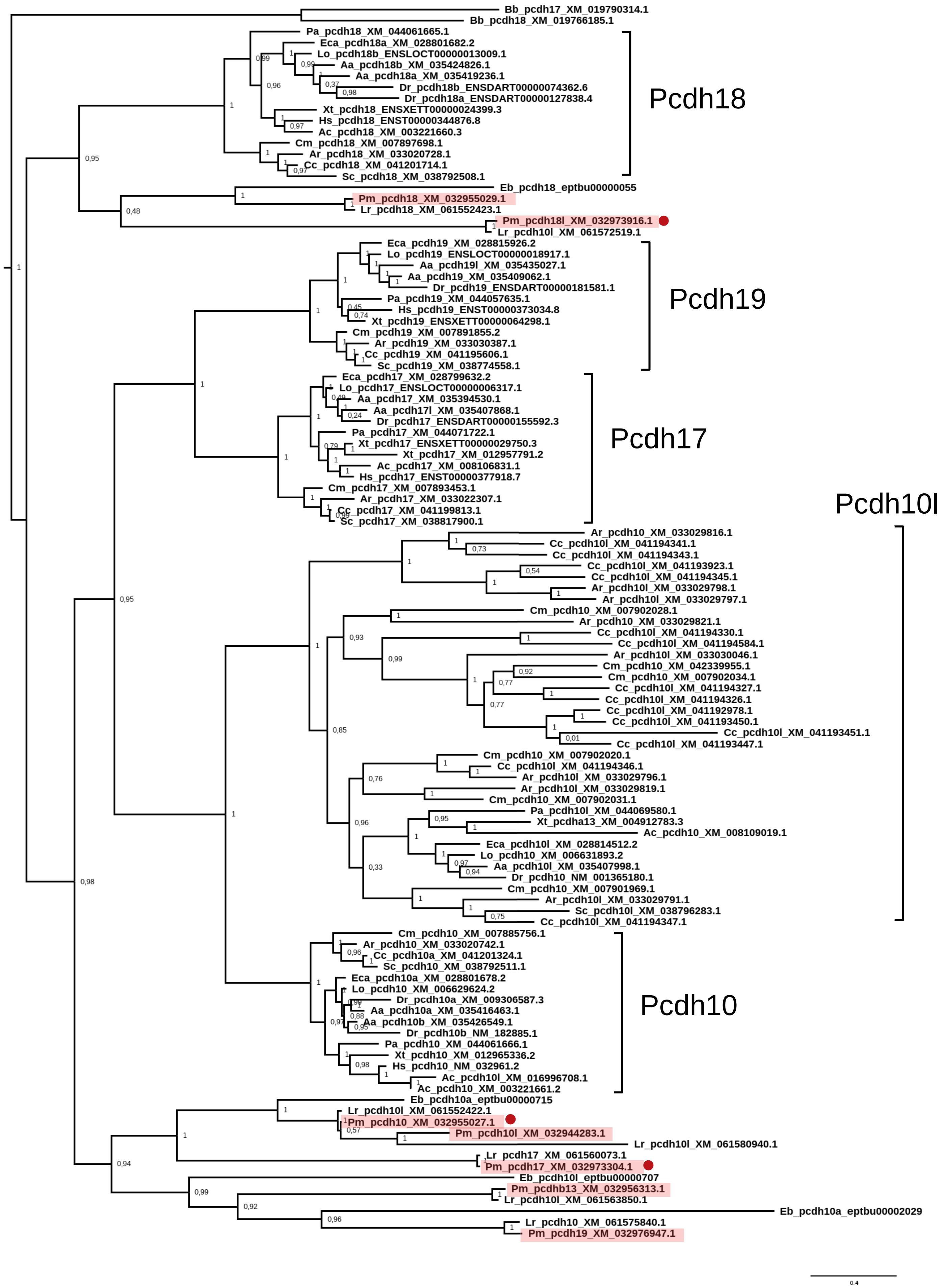

Supplementary Figure 18

continued on next page

b *Sox1/2/3/19* family

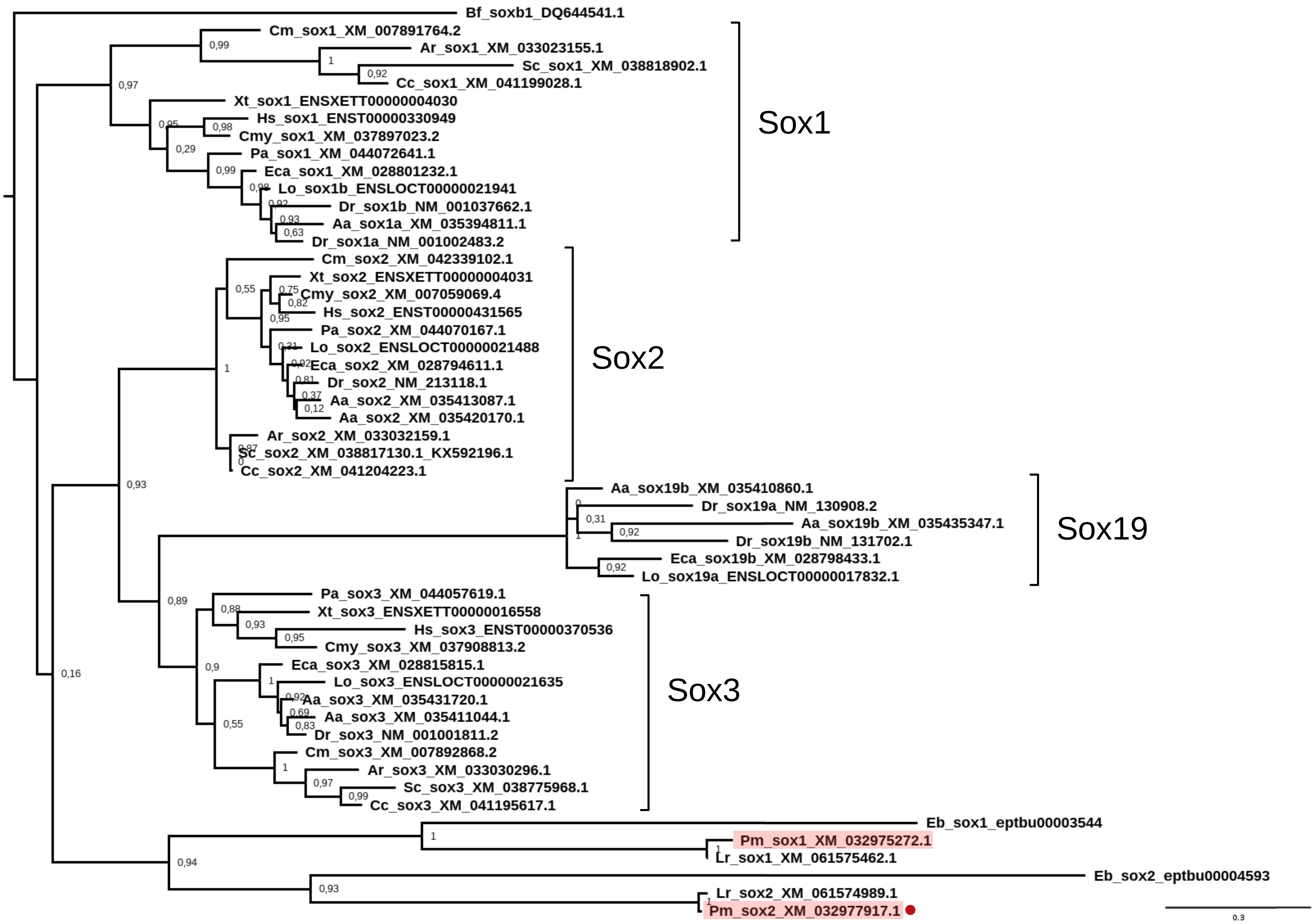

Supplementary Figure 18

continued on next page

C *Ntng1/2* family

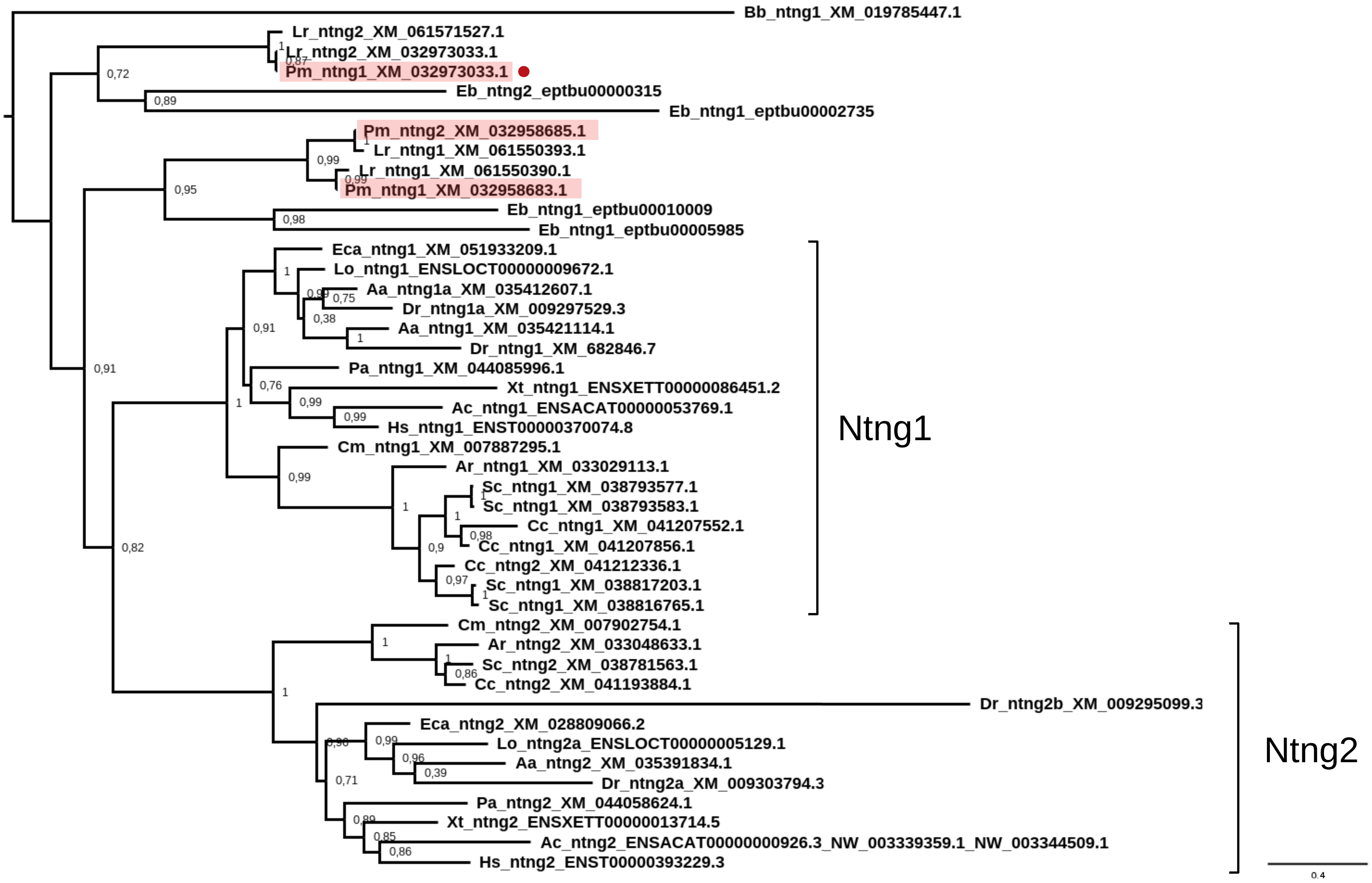

Supplementary Figure 18

continued on next page

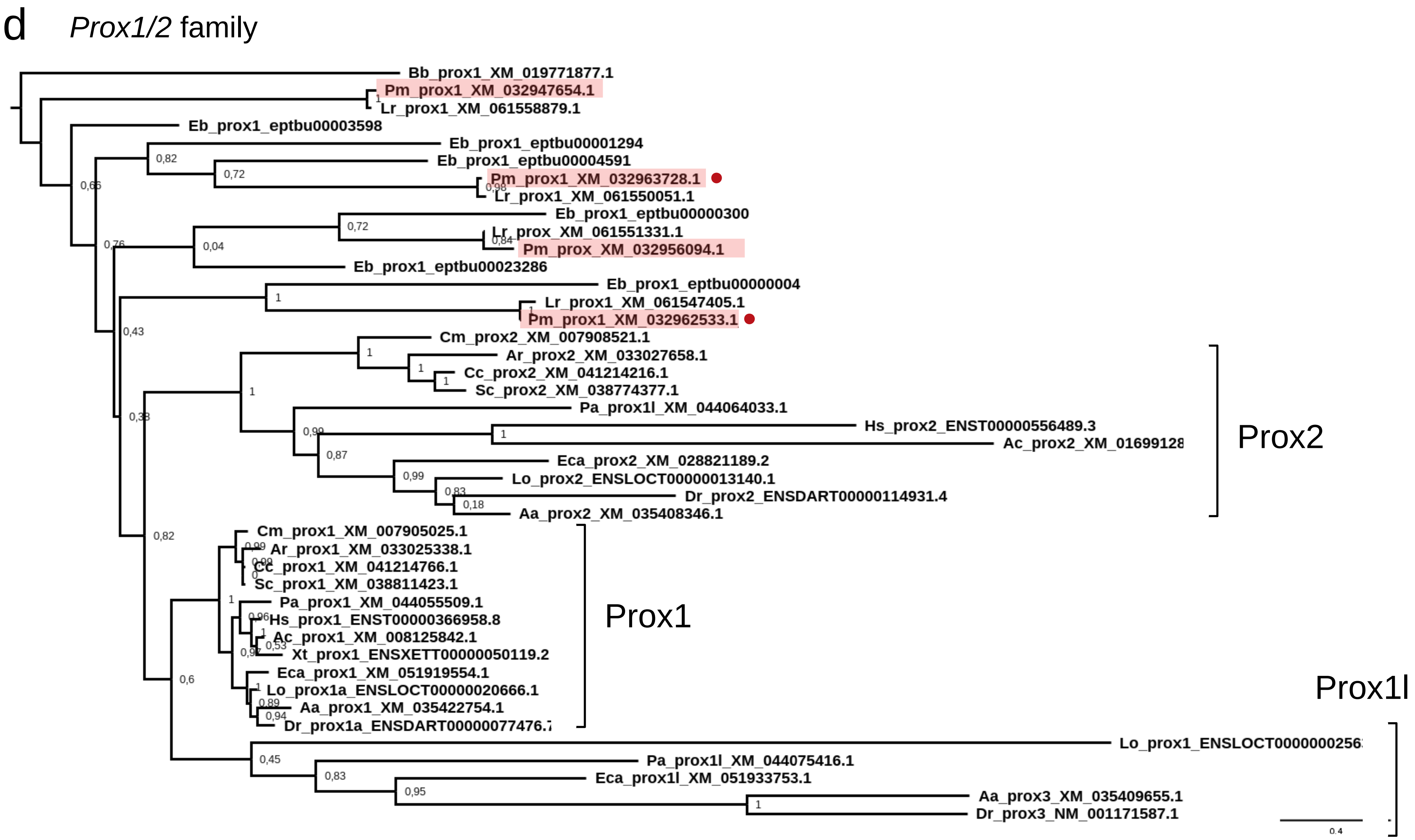

Supplementary Figure 18

continued on next page

e *Kctd8/12/12a/16* family

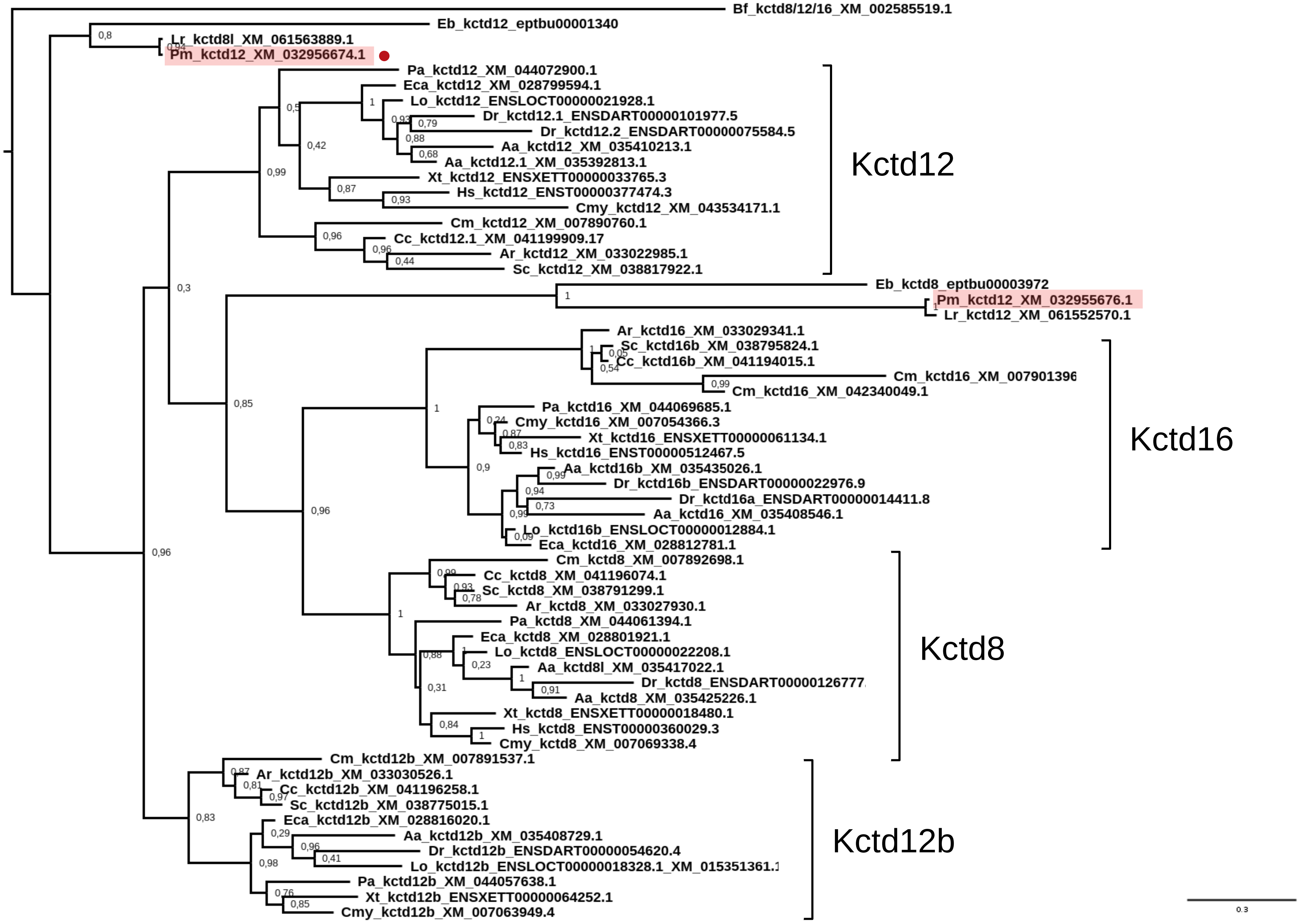

Supplementary Figure 18

See legend on next page

Supplementary Figure 18. Maximum likelihood phylogenetic trees showing gene content and relationships within the vertebrate *Pcdh10/10l/17/18/19* (a), *Sox1/2/3/19* (b), *Ntng1/2* (c), *Prox1/2* (d), and *Kctd8/12/16* (e) gene families. For each family and each species considered, the full repertoire of genes was retrieved by similarity search from genomic databases. Genes are identified in the trees by their NCBI or Ensembl identifier. The trees were rooted using the amphioxus representative of the family as outgroup. The trees were calculated using PhyML with the following parameters: NNI moves, LG+R4, aLRT branch support. The numbers at each node indicate the posterior probability of group occurrence. Gnathostome orthology classes are delineated by brackets on the right. Genes identified in *Petromyzon marinus* are shaded in red. A red dot indicates those, whose orthologs were analyzed by ISH in the river lamprey. Species abbreviations: Aa, *Anguilla anguilla*; Ac, *Anolis carolinensis*; Ar, *Amblyraja radiata*; Bf, *Branchiostoma floridae*; Bb, *Branchiostoma belcheri*; Cc, *Carcharodon carcharias*; Cm, *Callorhinchus milii*; Cmy, *Chelonia mydas*; Dr, *Danio rerio*; Eb, *Eptatretus burgeri*; Eca, *Erpetoichtys calabaricus*; Hs, *Homo sapiens*; Lo, *Lepisosteus oculatus*; Lr, *Lampetra reissneri*; Pm, *Petromyzon marinus*; Sc, *Scyliorhinus canicula*; Xt, *Xenopus tropicalis*.

	Catshark	Mouse	Zebrafish
Sox1	Left-LHb	LHb (LacZ reporter in *Sox1-LacZ*-knock-in line): *Kan et al. 2007*	Hb11; Ad_Hb11 (*sox1a/b*): *Pandey et al. 2018*
Pcdh17		LHb, PVT: *Allen Brain Atlas*	-
Lhpfl5		-	-
Ntng2		LHb, MHb: *Allen Brain Atlas*	-
Ptprm		-	-
Spon1	External-MHb anterior left	lateral MHb: *Allen Brain Atlas*	dHb, vHb (*spon1b*): *deCarvalho et al. 2014*; Hb07 (*spon1a*): *Pandey et al. 2018*
Stk32c		-	-
Trhde		lateral MHb: *Allen Brain Atlas*	-
Trhr2		MHb: *Heuer et al. 2000*	-
Pde1a		MHb: *Allen Brain Atlas*	-
Kctd12a		MHb: *Metz et al. 2011*	Ad_Hb04/06 (*kctd12.1, lov*), Ad_Hb02B (*kctd12.2, ron*): *Gamse et al, 2003*; *Gamse et al. 2005*; *Aizawa et al. 2007*; *Pandey et al. 2018*
Kctd12b	MHb	MHb: *Metz et al. 2011*	-
Kctd8		MHb: *Allen Brain Atlas, Metz et al. 2011*	dHb, right-enriched (*Kctd8,dex*): *Gamse et al. 2005*
Nrp2		Hb: *Allen Brain Atlas*	-
Stac1		MHb: *Allen Brain Atlas*	-
Enpp2	External MHb, right + posterior left	-	-
Eya4		-	-
Ak5	Right-LHb	-	Hb15; Ad_VHb01 (*ak5*): *Pandey et al. 2018*
Kiss1		-	vHb: *deCarvalho et al. 2014*; Hb15; Ad_VHb01 (*kiss1*): *Pandey et al. 2018*
Prox1		PVT: *Allen Brain Atlas*	Hb01 (*prox1a*): *Pandey et al. 2018*
Rerg		PVT: *Allen Brain Atlas*	Ad_VHb02/03/04: *Pandey et al. 2018*
Stxbp6		Thalamic nuclei adjacent to Hb, including PVT: *Allen Brain Atlas*	-
Prkcq		MHb+dispersed LHb cells: *Allen Brain Atlas*	Hb15 (*prkcq*): *Pandey et al. 2018*
Wisp1		-	-
Gng14		-	Hb15; Ad_VHb01 (si:dkey-117i10.1): *Pandey et al. 2018*
Shh		-	-
Trib1		-	-

Supplementary Table 1. Expression characteristics of mouse and zebrafish orthologs of markers of the main habenular territories identified in the catshark. *(legend on next page)*

Supplementary Table 1. Expression characteristics of mouse and zebrafish orthologs of markers of the main habenular territories identified in the catshark. Markers of the broad territories identified in catshark habenulae are listed in the first column, with their expression site in the second column. Expression territories of their orthologs in mouse habenulae or adjacent thalamic territories are shown in the third column, based on references cited or searches in the Allen Brain Atlas. The fourth column shows zebrafish orthologs, which were identified as signatures of cell clusters in a single-cell RNA-seq characterization of zebrafish habenulae (Pandey et al. 2018). The identity of cell clusters is indicated and their habenular location is the following: Hb11 and Ad_Hb11, larval and related adult ventral cell clusters; Hb07, left-enriched dorsal cell cluster; Ad-Hb02B/04/06, adult cell clusters related to larval dorsal habenula cell clusters (right-enriched for Ad_Hb02B); Hb15, larval ventral cell cluster; Hb01, larval dorsal right enriched cell cluster; Ad_VHb01/02/03/04, adult ventral cell clusters. References cited: Aizawa et al. 2007. Dev. Cell 12:87-98; Allen Brain Atlas, Lein et al. 2007. Nature 445:168-176; deCarvalho et al. 2014. Genesis 52:636-655; Gamse et al. 2003. Development 130:1059-1068; Gamse et al. 2005. Development 132:4869-4881; Heuer et al. 2000. J. Comp. Neurol. 428:319-336; Green et al. 2023. Biol. Psychiatry Glob. Open Sci 3:686-697; Kan et al. 2007. Dev. Biol. 310:85-98; Metz et al. 2011. J. Comp. Neurol. 519:1435-1454; Pandey et al. 2018. Curr. Biol. 8:1052-1065.e7.

	Control embryos		IWR-1-treated embryos	
Phenotype / Marker	Same phenotype as untreated embryos	Presence of zones of Left-LHb identity on the right	Same phenotype as untreated embryos	Presence of zones of Left-LHb identity on the right
ScSox1	7/7	0/7	0/9	9/9
ScNtng2	4/4	0/4	0/5	5/5
β–catenin	5/5	0/5	0/6	6/6
ScProx1	6/6	0/6	0/8	8/8
ScKiss1	3/3	0/3	0/3	3/3
ScRorα	3/3	0/3	0/3	3/3
Total number of embryos	8/8	0/8	0/9	9/9

Supplementary Table 2. Asymmetry phenotypes in lateral habenulae of control and IWR-1-treated catshark embryos. Phenotypes were analyzed at stage 31, following treatments as described in the Methods section. For each marker listed in the first column, the ratio shown refers to the number of embryos devoid of, or exhibiting zones of Left-LHb identity on the right, compared to the number of embryos analyzed. For *ScSox1* and *ScProx1*, embryo counts take into account phenotypes observed after detection both by chromogenic ISH and HCR-FISH. The last line indicates the total number of embryos analyzed.

	SB-505124-treated embryos embryos		SB-505124-+IWR-1-treated embryos	
Phenotype / Marker	Right isomerism	Presence of bilateral zones of Left-LHb identity	Right isomerism	Presence of bilateral zones of Left-LHb identity
ScSox1	5/5	0/5	0/9	9/9
ScNtng2	3/3	0/3	0/5	5/5
β–catenin	3/3	0/3	0/5	5/5
ScProx1	5/5	0/5	0/9	9/9
ScKiss1	3/3	0/3	0/5	5/5
Total number of embryos	6/6	0/6	0/10	10/10

Supplementary Table 3. Asymmetry phenotypes in lateral habenulae of SB-505124- and SB-505124-+IWR-1-treated catshark embryos. Phenotypes were analyzed at stage 31, following treatments as described in the Methods section. For each marker listed in the first column, the ratio shown refers to the number of embryos showing a right isomerism, or exhibiting bilateral zones of Left-LHb identity, compared to the number of embryos analyzed. For *ScSox1* and *ScProx1*, embryo counts take into account phenotypes observed after detection both by chromogenic ISH and HCR-FISH. The last line indicates the total number of embryos analyzed.

Gnathostome gene family	*P. marinus* gene NCBI Id	*P. marinus* gene Id (Lamanna et al. 2023)	Habenular clusters PCT>0.5	Parapineal clusters PCT>0.5	Pineal clusters PCT>0.5
Pcdh10/10l/ 17/18/19	XM_032973916.1 (*Pcdh18l*)	ND	ND	ND	ND
	XM_032955029.1	MSTRG.8598	-	-	-
	XM_032956313.1	MSTRG.7359	-	-	-
	XM_032976947.1	MSTRG.3986	-	-	-
	XM_032955027.1 (*Pcdh10l*)	PMZ-0037121	-	-	-
	XM_032944280.1	PMZ-0038866	-	-	-
	XM_032944283.1	PMZ-0038866	-	-	-
	XM_032973304.1 (*Pcdh17l*)	MSTRG.1202	DeChol1 (0.78)<br>DeChol2 (0.71)<br>DeChol3 (0.69)<br>DeChol4 (0.83)<br>DeExc1 (0.64)<br>DeExc2 (0.87)<br>DeExc3 (0.71)	Photo4 (0.73)	Photo2 (0.68)
Sox1/2/3/19	XM_032975272.1 (*Sox1l*)	MSTRG.14102	-	Photo3 (0.95)<br>Photo4 (0.79)	-
	XM_032977917.1 (*Sox2l*)	MSTRG.15764	-	-	-
Ntng1/2	XM_032973033.1 (*Ntng2l*)	MSTRG.10512	DeChol1 (0.86)<br>DeChol2 (0.74)<br>DeChol3 (0.69)<br>DeChol4 (0.83)<br>DeExc2 (0.67)<br>DeExc3 (0.71)	Photo3 (1.0)<br>Photo4 (0.96)	Photo1 (0.87)
	XM_032958683.1 (*Ntng1l*)	MSTRG.8166<br>MSTRG.18063	-<br>-	Photo3 (0.77)<br>-	Photo2 (0.81)<br>-
	XM_032958685.1 (*Ntng1l*)	MSTRG.18065	-	-	Photo2 (0.81)
Prox1/2	XM_032947654.1	MSTRG.20120	-	-	-
	XM_032956094.1	MSTRG.8252<br>MSTRG.8253	-<br>-	-<br>-	-<br>-
	XM_032963728.1 (*Prox1lb*)	MSTRG.17410	DeChol1 (0.59)<br>DeChol2 (0.61)<br>DeChol3 (0.54)	-	-
	XM_032962533.1 (*Prox1la*)	ND	ND	ND	ND
Kctd8/12/16	XM_032956674.1 (*Kctd12l*)	MSTRG.7325	DeChol1 (0.86)<br>DeChol2 (0.68)<br>DeChol3 (0.65)<br>DeExc1 (0.98)<br>DeExc2 (0.97)<br>DeExc3 (0.96)	-	-
	XM_032955676.1	MSTRG.8686	-	-	-

Supplementary Table 4. *(legend on next page)*

Supplementary Table 4. Expression of lamprey (*Petromyzon marinus*) members of the *Pcdh10/10l/17/18/19*, *Sox1/2/3/19*, *Ntng1/2*, *Prox1/2*, and *Kctd8/12/16* gene families in habenula, pineal and parapineal cell clusters, as inferred from a single-nuclei RNA-seq analysis of the lamprey brain. For each gene family (1st column), the lamprey paralogs are listed, with their NCBI identifier (2nd column) and the corresponding identifier used in Lamanna et al. 2023. Nat. Ecol. Evol. 7:1741 (3rd column). The percentage of expressing cells (PCT) in cell clusters identified as part of habenulae (DeChol-4, DeExc1-3), parapineal (Photo3,4) and pineal (Photo1,2) is indicated between brackets for each gene in the 4th, 5th and 6th columns, respectively. This analysis was conducted using the atlas available at: https://apps.kaessmannlab.org/adult_dien/. Only PCT>0.5 were taken into account. Paralogs submitted to ISH analysis are shown in red characters.
