# Supplementary material for: Analysis of a shark reveals ancient, Wnt-dependent, habenular asymmetries in vertebrates
Source: Nat Commun. 2024 Nov 25;15:10194. doi: 10.1038/s41467-024-54042-2 (PMC11589584; doi:10.1038/s41467-024-54042-2)
Supplement: Supplementary file 3 — Description of Additional Supplementary Files [file 41467_2024_54042_MOESM3_ESM.pdf]

Description of Additional Supplementary Files

File Name: Supplementary Data 1

Description: Transcriptomic analysis of habenular asymmetries in catshark stage 31 habenulae. Sheet 1 List of differentially expressed genes between left and the right habenulae. Differentially expressed gene models are listed in column A, with left- and right-enriched ones shaded in yellow and blue respectively. Contigs exhibiting statistically significant count differences between the left and right habenulae were identified using a two-sided Wald test implemented in sleuth (Pimentel et al., 2017. Nat Methods. Jul;14(7):687-690; q-value threshold 5E-02). Curated annotation and automated annotation against Swissprot are indicated in columns B and C. p-values, q-values and fold changes (FC) are given in columns D, E and G. Genes previously identified as left-enriched in developing catshark habenulae (Lagadec et al, 2015. Nat. Commun. 6, 6686) such as *ScPitx2* and *ScKctd12b*, are present in this list. Sheet 2 Functional annotation of left- and right enriched genes. GO (gene ontology) terms for biological processes over-represented in the lists of left- and right-enriched genes are represented in columns (B,C) and (G,H), respectively, with the corresponding q-values in A and F and the identity of genes in D and I. Terms are grouped together by level (2 to 5). Those retrieved both for left- and right-enriched genes are shown in red characters.

File Name: Supplementary Data 2

Description: List of genes analyzed by ISH in the catshark *Scyliorhinus canicula*, the elephant shark *Callorhinchus milii*, the reedfish *Erpetoichthys calabaricus*, the spotted gar *Lepisosteus oculatus*, the West African lungfish *Protopterus annectens*, the Western clawed frog Xenopus tropicalis, and the river lamprey *Lampetra fluviatilis*. Sheet 1 The genes shown were retrieved from the transcriptomic comparison between stage 31 left and right habenulae except for *ScKctd8*, *ScKctd12a* and *ScRora*, which were selected by candidate gene approaches as described in the Results section. Gene names are indicated in column A, the corresponding identification in the reference database in column B, the laterality of the enrichment predicted by the transcriptomic analysis in column C, the rank in the list of differentially expressed genes in column D (ranking on increasing p-values), the corresponding expression characteristics in column E and the sequence of the probe in column F. Colors indicate the expression characteristics of genes exhibiting regionalized ISH in profiles in differentiated habenula territories with the following code: yellow, Left-LHb; light purple, MHb; dark purple, external MHb, right and posterior-left; magenta, external MHb, anterior-left; blue, Right-LHb). Genes expressed in combinations of these domains are shaded in grey. Sheet 2-6 Columns A, B and C, respectively, contain gene names, their NCBI identifier, and probe sequences. Sheet 7 Columns A, B, C and D, respectively, contain gene names, the NCBI identifier of the marine lamprey ortholog, the identifier of the river lamprey ortholog when available, and probe sequences. Probe sequences were obtained from river lamprey sequences, except for *LfPcdh18l* (line 1) and the two *Kiss* genes (lines 10 and 11), where *P. marinus* sequences were used. The two species exhibit a high level of similarity in their coding sequences, allowing extensive cross-hybridization signals (Lagadec et al. 2015. Nature Com. 6:6686)
