# Supplementary material for: Analysis of a shark reveals ancient, Wnt-dependent, habenular asymmetries in vertebrates
Source: Nat Commun. 2024 Nov 25;15:10194. doi: 10.1038/s41467-024-54042-2 (PMC11589584; doi:10.1038/s41467-024-54042-2)
Supplement: Supplementary file 6 — Reporting Summary [file 41467_2024_54042_MOESM6_ESM.pdf]

nature portfolio

Corresponding author(s): Sylvie Mazan

Last updated by author(s): Oct 16, 2024

Reporting Summary

Nature Portfolio wishes to improve the reproducibility of the work that we publish. This form provides structure for consistency and transparency in reporting. For further information on Nature Portfolio policies, see our Editorial Policies and the Editorial Policy Checklist.

Please do not complete any field with "not applicable" or n/a. Refer to the help text for what text to use if an item is not relevant to your study.
For final submission: please carefully check your responses for accuracy; you will not be able to make changes later.

Statistics

For all statistical analyses, confirm that the following items are present in the figure legend, table legend, main text, or Methods section.

n/a	Confirmed	
☐	☒	The exact sample size (*n*) for each experimental group/condition, given as a discrete number and unit of measurement
☐	☒	A statement on whether measurements were taken from distinct samples or whether the same sample was measured repeatedly
☐	☒	The statistical test(s) used AND whether they are one- or two-sided<br>*Only common tests should be described solely by name; describe more complex techniques in the Methods section.*
☒	☐	A description of all covariates tested
☒	☐	A description of any assumptions or corrections, such as tests of normality and adjustment for multiple comparisons
☒	☐	A full description of the statistical parameters including central tendency (e.g. means) or other basic estimates (e.g. regression coefficient) AND variation (e.g. standard deviation) or associated estimates of uncertainty (e.g. confidence intervals)
☐	☒	For null hypothesis testing, the test statistic (e.g. F, t, r) with confidence intervals, effect sizes, degrees of freedom and P value noted<br>*Give P values as exact values whenever suitable.*
☒	☐	For Bayesian analysis, information on the choice of priors and Markov chain Monte Carlo settings
☒	☐	For hierarchical and complex designs, identification of the appropriate level for tests and full reporting of outcomes
☒	☐	Estimates of effect sizes (e.g. Cohen's d, Pearson's r), indicating how they were calculated

Our web collection on statistics for biologists contains articles on many of the points above.

Software and code

Policy information about availability of computer code

Data collection	Confocal images were acquired using an inverted Leica SP8 microscope (Leica Microsystems Inc. Wetzlar, Germany) equipped with SuperK EXTREME white laser source (NKT Photonics A/S, Birkerød, Denmark) and Leica Hybrid Detector and Leica Application Suite X (LAS X) software (version 3.5.7.23225).<br>Brightfleld images were acquired by Zeiss Axioscope 5 (Carl Zeiss Microscopy, Germany) equipped with ZEISS Axiocam 208 color and Zeiss ZEN Blue software (version 3.7.4)
Data analysis	The algorithms used (kallisto version 0.46.1;sleuth; Fiji) were previously published and references are provided.

For manuscripts utilizing custom algorithms or software that are central to the research but not yet described in published literature, software must be made available to editors and reviewers. We strongly encourage code deposition in a community repository (e.g. GitHub). See the Nature Portfolio guidelines for submitting code & software for further information.

Data

Policy information about availability of data

All manuscripts must include a data availability statement. This statement should provide the following information, where applicable:

- Accession codes, unique identifiers, or web links for publicly available datasets
- A description of any restrictions on data availability
- For clinical datasets or third party data, please ensure that the statement adheres to our policy

All data supporting the findings of this study are available within the paper and its Supplementary Information. Transcriptomic data were deposited into the Sequence Read Archive (SRA) NCBI database under accession number PRJNA1040760 and are available at the following URL: https://www.ncbi.nlm.nih.gov/sra/?term=PRJNA1040760. River lamprey sequences were deposited in the NCBI nucleotide database and are available under numbers PQ241090, PQ241091,PQ241092,PQ241093,PQ241094,PQ241095 and PQ241096.

Research involving human participants, their data, or biological material

Policy information about studies with human participants or human data. See also policy information about sex, gender (identity/presentation), and sexual orientation and race, ethnicity and racism.

Reporting on sex and gender	N/A
Reporting on race, ethnicity, or other socially relevant groupings	N/A
Population characteristics	N/A
Recruitment	N/A
Ethics oversight	N/A

Note that full information on the approval of the study protocol must also be provided in the manuscript.

Field-specific reporting

Please select the one below that is the best fit for your research. If you are not sure, read the appropriate sections before making your selection.

☒ Life sciences ☐ Behavioural & social sciences ☐ Ecological, evolutionary & environmental sciences

Life sciences study design

All studies must disclose on these points even when the disclosure is negative.

Sample size	No sample-size calculation was performed. For RNA-seq experiment 3 pools of habenulae were prepared. Each pool contains 15 left habenulae or their right counterpart. 45 individuals were chosen in order to have enough material and limit interindividual variation.<br>For experimental analyses in the catshark S. canicula, the availability of embryos is a limiting factor due to (i) seasonal reproduction, (ii) the low reproduction rate (about two eggs every two weeks per female during the Spring, which corresponds to the peak of the spawning season and (iii) the relatively large size of adults, precluding the maintenance of large breeder cohorts in laboratory facilities. However, for in ovo drug treatments, a minimum of six biological replicates (different specimens) were used per condition and the penetrance of the asymmetry phenotypes reported was 100% in all cases. For BrdU incorporation tests, a minimum of three independent specimens were used for each incorporation stage analyzed, with the same results.<br>For ISH and IHC, some specimens are relatively difficult to obtain (lungfish, reedfish) but for every marker analyzed, a minimum of three specimens were used, and results identical to the one shown were consistently obtained. The number of sample analyzed is included in the Figure legends.
Data exclusions	No data were excluded from the analyses.
Replication	RNA-seq analyses were performed in triplicates. All attempts at replication were successful.
Randomization	Embryos at the desired developmental stages were chosen randomly from the same aquarium.
Blinding	Blinding was not relevant to this study because experimental and control conditions were performed in parallel.

Reporting for specific materials, systems and methods

We require information from authors about some types of materials, experimental systems and methods used in many studies. Here, indicate whether each material, system or method listed is relevant to your study. If you are not sure if a list item applies to your research, read the appropriate section before selecting a response.

Materials & experimental systems

n/a	Involved in the study	
☐	☒	Antibodies
☒	☐	Eukaryotic cell lines
☒	☐	Palaeontology and archaeology
☐	☒	Animals and other organisms
☒	☐	Clinical data
☒	☐	Dual use research of concern
☒	☐	Plants

Methods

n/a	Involved in the study	
☒	☐	ChIP-seq
☒	☐	Flow cytometry
☒	☐	MRI-based neuroimaging

Antibodies

Antibodies used	β-catenin (Ab6302, 1/1000, Abcam, Boston, MA, USA)<br>HuC/D (A-21271, 1/400, Molecular Probes)<br>Acetylated tubulin (T-6793, 1/200, Sigma-Aldrich, Saint-Louis, MO, USA)<br>anti-BrdU primary antibody (sc-32323, 1/100, Santa Cruz Biotechnology, Dallas, TX, USA)
Validation	No validation statements for commercially available antibodies included here as all primary antibodies are validated by the vendors.

Animals and other research organisms

Policy information about studies involving animals; ARRIVE guidelines recommended for reporting animal research, and Sex and Gender in Research

Laboratory animals	This study involed Xenopus tropicalis tadpoles NF66
Wild animals	Juveniles of Protopterus annectens, Lepisosteus oculatus and Erpetoichthys calabaricus were purchased from commercial sources and euthanized immediately with an overdose of tricaine.<br>Scyliorhinus canicula and Lampetra fluviatilis were provided by professional fishermen, transported (Type 1 agreement for living animals transportation number 66082) to, and maintained by the Aquariology Service of the Banyuls-sur-Mer Oceanological Observatory (agreement number A6601602)<br>Callorhinchus milii females were caught by rod and reel from Western Port Bay, Victoria, Australia (approved by the Department of Primary Industries, Victoria, Australia, permit DPI RP1000, RP1003, and RP1112) and all procedures were approved by Monash University Animal Ethics (Ethics license permit MAS/ARMI/2010/01)
Reporting on sex	No sex based analysis were performed.
Field-collected samples	The study involved no experimental work on field-collected samples, but only on catshark eggs, collected in the Banyuls-sur-Mer Oceanological Observatory. Only tissue (brain) collection was conducted on other specimens.
Ethics oversight	Ethical review and approval were not required for this project, because this study only used tissue collection from euthanized specimens or experimentation on non-mammalian, non-free-feeding embryos. This work thus does not require approval from an animal ethics committee according to French and European regulations

Note that full information on the approval of the study protocol must also be provided in the manuscript.

Plants

Seed stocks	N/A
Novel plant genotypes	N/A
Authentication	N/A

This checklist template is licensed under a Creative Commons Attribution 4.0 International License, which permits use, sharing, adaptation, distribution and reproduction in any medium or format, as long as you give appropriate credit to the original author(s) and the source, provide a link to the Creative Commons license, and indicate if changes were made. The images or other third party material in this article are included in the article's Creative Commons license, unless indicated otherwise in a credit line to the material. If material is not included in the article's Creative Commons license and your intended use is not permitted by statutory regulation or exceeds the permitted use, you will need to obtain permission directly from the copyright holder. To view a copy of this license, visit http://creativecommons.org/licenses/by/4.0/
